# mTOR/EGFR/iNOS/MAP2K1/FGFR/TGFB1 Are Druggable Candidates for N-(2,4-Difluorophenyl)-2′,4′-Difluoro-4-Hydroxybiphenyl-3-Carboxamide (NSC765598), With Consequent Anticancer Implications

**DOI:** 10.3389/fonc.2021.656738

**Published:** 2021-03-26

**Authors:** Bashir Lawal, Ching-Yu Lee, Ntlotlang Mokgautsi, Maryam Rachmawati Sumitra, Harshita Khedkar, Alexander T.H. Wu, Hsu-Shan Huang

**Affiliations:** ^1^ PhD Program for Cancer Molecular Biology and Drug Discovery, College of Medical Science and Technology, Taipei Medical University and Academia Sinica, Taipei, Taiwan; ^2^ Graduate Institute for Cancer Biology & Drug Discovery, College of Medical Science and Technology, Taipei Medical University, Taipei, Taiwan; ^3^ Department of Orthopedics, Taipei Medical University Hospital, Taipei, Taiwan; ^4^ Department of Orthopaedics, School of Medicine, College of Medicine, Taipei Medical University, Taipei, Taiwan; ^5^ TMU Research Center of Cancer Translational Medicine, Taipei Medical University, Taipei, Taiwan; ^6^ The PhD Program of Translational Medicine, College of Science and Technology, Taipei Medical University, Taipei, Taiwan; ^7^ Clinical Research Center, Taipei Medical University Hospital, Taipei Medical University, Taipei, Taiwan; ^8^ Graduate Institute of Medical Sciences, National Defense Medical Center, Taipei, Taiwan; ^9^ School of Pharmacy, National Defense Medical Center, Taipei, Taiwan; ^10^ PhD Program in Biotechnology Research and Development, College of Pharmacy, Taipei Medical University, Taipei, Taiwan

**Keywords:** NSC765598, multi-omics study, multi-target small molecule, anticancer activity, molecular docking, protein-ligand interaction

## Abstract

**Background:**

The application of computational and multi-omics approaches has aided our understanding of carcinogenesis and the development of therapeutic strategies. NSC765598 is a novel small molecule derivative of salicylanilide. This study aims to investigate the ligand-protein interactions of NSC765598 with its potential targets and to evaluate its anticancer activities *in vitro.*

**Methods:**

We used multi-computational tools and clinical databases, respectively, to identify the potential drug target for NSC765598 and analyze the genetic profile and prognostic relevance of the targets in multiple cancers. We evaluated the *in vitro* anticancer activities against the National Cancer Institute 60 (NCI60) human tumor cell lines and used molecular docking to study the ligand-protein interactions. Finally, we used the DTP-COMPARE algorithm to compare the NSC765598 anticancer fingerprints with NCI standard agents.

**Results:**

We identified mammalian target of rapamycin (mTOR)/epidermal growth factor receptor (EGFR)/inducible nitric oxide synthase (iNOS)/mitogen-activated protein 2 kinase 1 (MAP2K1)/fibroblast growth factor receptor (FGFR)/transforming growth factor-β1 (TGFB1) as potential targets for NSC765598. The targets were enriched in cancer-associated pathways, were overexpressed and were of prognostic relevance in multiple cancers. Among the identified targets, genetic alterations occurred most frequently in EGFR (7%), particularly in glioblastoma, esophageal squamous cell cancer, head and neck squamous cell cancer, and non–small-cell lung cancer, and were associated with poor prognoses and survival of patients, while other targets were less frequently altered. NSC765598 displayed selective antiproliferative and cytotoxic preferences for NSCLC (50% growth inhibition (GI_50_) = 1.12–3.95 µM; total growth inhibition (TGI) = 3.72–16.60 μM), leukemia (GI_50_ = 1.20–3.10 µM; TGI = 3.90–12.70 μM), melanoma (GI_50_ = 1.45–3.59 µM), and renal cancer (GI_50_ = 1.38–3.40 µM; TGI = 4.84–13.70 μM) cell lines, while panels of colon, breast, ovarian, prostate, and central nervous system (CNS) cancer cell lines were less sensitive to NSC765598. Interestingly, NSC765598 docked well into the binding cavity of the targets by conventional H-bonds, van der Waal forces, and a variety of π-interactions, with higher preferences for EGFR (ΔG = −11.0 kcal/mol), NOS2 (ΔG = −11.0 kcal/mol), and mTOR (ΔG = −8.8 kcal/mol). NSC765598 shares similar anti-cancer fingerprints with NCI standard agents displayed acceptable physicochemical values and met the criteria of drug-likeness.

**Conclusion:**

NSC765598 displayed significant anticancer and potential multi-target properties, thus serve as a novel candidate worthy of further preclinical studies.

## Introduction

Currently, cancer is one of the leading causes of global morbidity and the second leading cause of mortality worldwide (1), accounting for an estimated 19.3 million new cases and 9.9 million deaths in 2020 (2). One in five men or women develops cancer, while death occurs in 1 out of 8 men or 1 out of 11 women. However, due to increasing rates of predisposing factors, such as smoking, a sedentary lifestyle, being overweight, and increasing pollution associated with urbanization and industrial development, cancer is predicted to top the rank of leading causes of global mortality and be the most important independent dictator of a poor life expectancy in the 21st century (3). In 2020, female breast cancer has surpassed lung cancer as the most commonly diagnosed cancer accounting for 11.7% (2.3 million new cases) (2), and closely followed by lung (11.7%), colorectal (10.0%), prostate (7.3%), and stomach (7.7%) cancers, whereas colorectal (9.4% deaths), liver (8.3% death), stomach (7.7% deaths), and breast (6.9% deaths) cancers followed lung cancer (with an estimated 1.8 million deaths (18%) in terms of mortality rates (2), with higher incidences in males than in females (4). Among women, breast cancer is the most frequently diagnosed and a prominent cause of cancer deaths, followed by colorectal, lung, and cervical cancers (3). Unfortunately, the global cancer burden is expected to be 28.4 million cases in 2040, a 47% rise from 2020 (2). This projection is solely due to the growth and aging of the population and an increasing prevalence of risk factors.

The most common strategy for managing cancer is conventional therapies involving surgery and chemo- and radiotherapies. However, despite the early effectiveness demonstrated by these oncological strategies (5, 6), therapeutic resistance and toxicity, among other factors, have limited the success rates and long-term survival of cancer patients. Hence, research is needed on novel therapeutic options, which can offer better and more-specific multi-target antitumor activities (7), with minimal side effects to ultimately enhance the survival of cancer patients. Without a doubt, multitarget small molecules with anticancer activities represent alternative strategies with promising features for the realization of novel drugs that could target multiple aberrant signaling pathways (8, 9), offering fewer chances for drug resistance and thus better prognoses of cancer patients.

Several potential molecular targets exist for exploring the dysregulation of signaling pathways of cancer cells (10–12). Growth factors, including epidermal growth factor (EGF), fibroblast growth factor (FGF), transforming growth factor (TGF), and vascular endothelial growth factor (VEGF), are compact molecules that play important roles in regulating cellular communication, growth and differentiation, proliferation, survival, migration, and metastasis of cancer cells (13, 14). Upon receptor binding, EGF and TGF activate downstream mitogen-activated protein kinase (MAPK) and AKT-mammalian target of rapamycin (mTOR) pathways (15), which regulate apoptosis and cell proliferation (16), while inducible nitric oxide synthase (iNOS) modulates cellular bioenergetics pathways in cancer (17). Therefore, these signaling pathways are important for cancer initiation, progression, and metastasis and thus act as attractive targets for anticancer therapy exploration. Mapping these targets with corresponding drugs, therefore, has become a relevant prerequisite for drug discovery.

Over the years, rational drug design (RDD) based on computational biology and bioinformatics has not only changed the way drugs are designed but also speed up drug discovery and development processes while reducing costs and the wasting of resources (18). Computational and bioinformatics studies of cancer involve a variety of methods including mining and analysis of clinical data to give insights into the prognostic relevance of gene signatures, analyze geno-phenotypic responses to clinical drugs, and identify novel compounds with their potential targets before *in vitro* and *in vivo* validation studies. One such method is molecular modeling and docking studies of receptor-ligand interactions. Molecular docking is frequently employed to predict binding orientations of small molecules with their target proteins in order to calculate the affinities and activities of the small-molecule drug candidates (18, 19).

In our previous study, a salicylanilide analogue, NDMC101 (PubChem CID: 60202556), was synthesized, and we observed significant cytotoxic effects *via* downregulation of proliferative and inflammatory markers (20). A series of modifications of the salicylanilide core scaffold of the lead molecule were synthesized, and their preclinical anticancer activities against a variety of cancers were reported (9, 21–23). As a continuing effort to find and screen more active derivatives of NDMC101, in the present study, we reported a new derivative, NSC765598, for anticancer activities and identified mTOR/EGR receptor (EGFR)/iNOS/MAP2K/TGF-β1 (TGFB1)/fibroblast growth factor receptor-1 (FGFR1) as its potential targets.

## Materials and Methods

### Drug Likeness, ADMET Properties, and Target Identification of NSC765598

**Figure 1:**
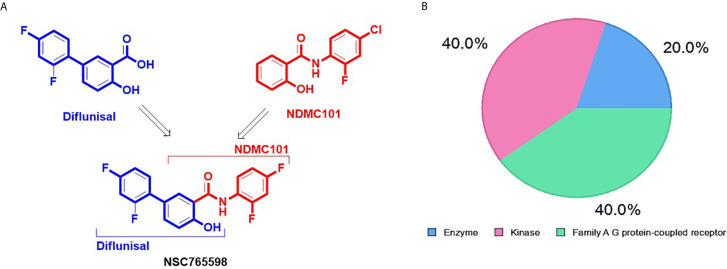
Synthetic protocol and potential drug target classification of N-(2,4-difluorophenyl)-2’,4’-difluoro-4-hydroxybiphenyl-3-carboxamide (NSC765598). **(A)** NSC765598 is a chemical derivative of diflunisal and NDMC101s (reaction conditions; SOCl_2_, anhydrous THF, reflux, 8 h; and aniline, anhydrous THF, reflux 14-16) **(B)**. The SwissTarget identified top 20 targets of NSC765598 are classified into enzymes (20%), kinases (40%) and Family A G protein-coupled receptor (40%).

NSC765598 was synthesized as a derivative of diflunisal and salicylanilides (NDMC101) (**Figure 1A**) *via* reactions (SOCl_2_, anhydrous THF, reflux, 8 h; and aniline, anhydrous THF, reflux 14-16) described in our previous study (21). We analyzed the drug-likeness, pharmacokinetics, and medicinal chemistry of NSC765598 using ADMETLab (http://admet.scbdd.com/), SwissADME software (http://www.swissadme.ch) (24), and the Lipinski’s rule-of-five algorithm (http://www.scfbio-iitd.res.in/software/drugdesign/lipinski.jsp) which identifies drug candidates based on the principle that drug candidates must have a molecular weight of < 500 Da, fewer than five hydrogen bond donors, fewer than ten hydrogen bond acceptors, the lipophilicity of < 5 LogP, and a molar refractivity range of 40 to 130 (25). NSC765598’s targets were identified using the PharmMapper Server (http://lilab-ecust.cn/pharmmapper/index.html) (26), SwissADME software, and computer-aided Prediction of Biological Activity Spectra (PASS) web resources (http://way2drug.com/dr) (27).

### Clinical Data Mining of Databases

Differential gene expression profiles of the EGFR, iNOS, mTOR, FGF receptor (FGFR), TGF receptor (TGFR), and MAP2K1 across The Cancer Genome Atlas (TCGA) databases were analyzed using the Tumor IMmune Estimation Resource (TIMER2.0) resource (http://timer.cistrome.org/) (28). The prognostic relevance of the genes in 9736 tumor samples across 33 TCGA cancer types was analyzed using the Gene Expression Profiling Interactive Analysis (GEPIA) (http://gepia.cancer-pku.cn/) (29). We collected the RNA expression profile of the genes and then set the median expression as the expression threshold to split the patient samples into high‐expression and low‐expression groups, and used the Kaplan‐Meier survival plot to assess the overall survival (OS) with the hazard ratio (HR), a 95% confidence interval (CI), and a log‐rank test p‐value. We also explored the cancer genomic dataset using the cBioPortal tool (http://www.cbioportal.org/) to analyze genomic alterations and survival and perform group comparisons of EGFR, iNOS, mTOR, FGFR, TGFR, and MAP2K1 in 10,953 cancer patients (10,967 samples) from different cancer types (30, 31).

### Protein-Protein Interactions (PPIs) and Functional Enrichment Analysis

PPI networks and functional enrichment analyses including Kyoto Encyclopedia of Genes and Genomes (KEGG) pathways and Gene Ontology (GO) biological processes of the selected NSC765598 target gene list were conducted using the Search Tool for Retrieval of Interacting Genes (STRING, vers. 10.5, (https://www.string-db.org/) with the adjusted threshold confidence set to 0.900 (32) and Enrich (https://maayanlab.cloud/Enrichr/enrich#) (33, 34). KEGG enrichment criteria were set to *p* < 10^−5^, gene count of ≥ 3, and strength of ≥ 2.

### Receptor-Ligand Interaction Study

The crystal structures of MAPK2K (PDB:3FXW), FGF (PDB: 1IJT), the mTORdeltaN-mLST8 complex (PDB: 4JSN), tumor growth factor beta1 (PDB:1KLC), EGFR kinase (T790M/L858R) apo (PDB: 5EDP), and iNOS (PDB:2BHJ) were obtained from the Protein Data Bank (https://www.rcsb.org/ ) in protein data bank (PDB) file format and subsequently converted into the Auto Dock Pdbqt format using AutoDock Vina (vers. 0.8, the Scripps Research Institute, La Jolla, CA, USA) (35). The three-dimensional (3D) structure of NSC765598 was built out with the Avogadro molecular builder and visualization tool vers. 1.XX (http://avogadro.cc/) (36). The structure was retrieved in mol2 file format and was subsequently transformed into PDB format using the PyMOL Molecular Graphics System, vers. 1.2r3pre (Schrödinger; https://pymol.org/edu/?q=educational/) followed by conversion to pdbqt. The three-dimensional (3D) structures of standard drugs; Dactolisib (CID: 11977753), Mirdametinib (CID: 9826528), Gefitinib (CID: 123631), N-Iminoethyl-l-lysine dihydrochloride (CID: 2733505), Erdafitinib (CID: 67462786), and Galunisertib (CID: 10090485) were retrieved in SDF file format from the PubChem database and subsequently converted to PDB format using the PyMOL tool and then to pdbqt format using the AutoDock Vina. Receptors were prepared by pre-docking removal of water (H_2_0) molecules, the addition of hydrogen atoms (polar only), and the addition of Kolmman charges (8). Molecular docking was performed using AutoDock Vina with all parameters set to default values, and all bonds in the ligand rotated freely while considering the receptor to be rigid. A grid box of 40 × 40 × 40 Å at X, Y, and Z dimensions and a spacing of 1.0 angstrom were used. All docking was performed at an exhaustiveness of 8. The docking outcome was analyzed in terms of the ligand’s affinity for the receptor and was expressed as binding energy values in Kcal/mol. The interactions in 2D conformation were visualized using the Discovery studio visualizer vers. 19.1.0.18287 (BIOVIA, San Diego, CA, USA) (37), while the hydrophobic contacts between the receptor-ligand complex were mapped out using the protein-ligand interaction profiler web tool (https://plip-tool.biotec.tu-dresden.de/plip-web/plip/index) (38).

### *In Vitro* Anticancer Screening of NSC765598 Against NCI60 Panels of Human Tumor Cell Lines

NSC765598 was screened for anti-proliferative and cytotoxic activities against NCI60 panels of cancer cell lines at the National Cancer Institute (NCI). Protocols for *in vitro* screening of bioactive compounds against NCI60 panels of cancer cell lines are well documented (39, 40). Briefly, about 5000 to 40,000 cells were seeded into each well of 96-well plates for 24 h. Cells were treated with a single dose (10 μM) of NSC765598 and incubated at 37°C, 5% CO_2_, 95% air, and 100% relative humidity for 48 h. Cell viability was determined using the sulforhodamine B (SRB) solution protocol (41). Following single-dose testing, NSC765598, was further evaluated for dose-dependent activities at five concentrations (0, 0.1, 1.0, 10, and 100 μM). Using the seven absorbance measurements [time zero, (Tz), control growth, (C), and test growth in the presence of drug at the five concentration (Ti)], the percentage growth is calculated at each of the drug concentrations levels. The percentage growth inhibition is calculated as: [(Ti-Tz)/(C-Tz)] × 100 for concentrations for which Ti>/=Tz or [(Ti-Tz)/Tz] × 100 for concentrations for which Ti<Tz.

Three dose-response parameters including the drug concentration causing total growth inhibition (TGI), drug concentration causing a 50% reduction in the net protein increase in control cells during the drug incubation (GI_50_), and drug concentration causing 50% cell lethality (LC_50_), indicating a net loss of cells following treatment) (42) were computed. GI_50_ is calculated from [(Ti-Tz)/(C-Tz)] × 100 = 50, TGI is calculated from Ti = Tz, while the LC_50_ is calculated from [(Ti-Tz)/Tz] × 100 = −50. Values are calculated for each of these three parameters if the level of activity is reached; however, if the effect is not reached or is exceeded, the value for that parameter is expressed as greater or less than the maximum or minimum concentration tested.

### DTP-COMPARE Analysis of NSC765598 Anticancer Fingerprints

The activity patterns (fingerprints) of NSC765598 were correlated to NCI synthetic compounds and standard agents using the DTP-COMPARE algorithms (43). The NSC numerical IDs were used as “seed” while GI_50_, TGI, and LC_50_ were set as the endpoints (8).

### Data Analysis and Visualization

Data were visualized using GraphPad Prism 8 software. Overall survival of the cohorts was visualized in Kaplan-Meier (K-M) survival plots. The statistical significance of differentially expressed genes was evaluated using the Wilcoxon test. *p<0.05; **p<0.01; and ***p<0.001 indicate statistical significance. Growth inhibition was calculated relative to cells without drug treatment and the time-zero control. The growth inhibition by NSC765598 in the single-dose assay was obtained by subtracting the positive value on the plot from 100, i.e., a value of 60 would indicate 40% growth inhibition.

## Results

### Drug-Likeness, Physicochemical, and ADMET Properties of NSC765598

NSC765598 was synthesized as a white powder purity of > 95%. Our evaluation of the drug- properties revealed that NSC765598 exhibited acceptable physicochemical and ADMET properties, and satisfied Lipinski’s rule of five for drug-likeness. It has good synthetic accessibility (2.27), a high bioavailability score (0.607–0.87), and low toxicity (LD_50_ = 880.766 mg/kg). Interestingly, NSC765598 exhibited a high human intestinal absorption capability and was permeant to the blood-brain barrier **(** **Table 1** **).**

**Table 1:** Drug likeness, medicinal chemistry, physicochemical, and ADMET properties of NSC765598.

Parameters	NSC765598	Reference Value
Formula	C_19_H_11_F_4_NO_2_	
Molecular weight	361.29 g/mol	150–500 g/mol
Fraction Csp3	0.00	0.25 to < 1
Num. rotatable bonds	4	
n-HBD	2	0–5
n-HBA	3	0–10
Molar Refractivity	87.769989	40–130
TPSA	49.33 Å²	20–130
Log *P*_o/w_ (XLOGP3)	4.867901	- 0.7 to 5
Consensus Log *P*_o/w_	5.09	
Log *S* (ESOL)	−6.08	0–6
HIA absorption	High (0.64)	
LogD_7.4_ (Distribution Coefficient D)	1.09	1–5
BBB permeant	Yes (0.831)	
Lipinski (Drug-likeness)	Yes (0.594)	
	20%	> 0.1
Bioavailability Score	Bioavailability; 0.87	
	30% Bioavailability;	
	0.607	
Synthetic accessibility	2.27	1 (very easy) to 10 (very difficult)
T _1/2_ (Half Life Time)	1.865 h	
CL (Clearance Rate)	1.49 ml/min/kg	
LD_50_ (LD_50_ of acute toxicity)	880.766 mg/kg	> 500 mg/kg
AMES (Ames Mutagenicity)	NO (0.234)	
SkinSen (Skin sensitization)	NO (0.436)	

TPSA, topological polar surface area; HIA, human intestinal absorption; n-HBA, Num. H-bond acceptors; n-HBD, Num. H-bond donors; BBB, blood brain barrier. Calculations were done using SwissADME and SwissTarget algorithms.

### EGFR, mTOR, NOS2, TGFB1, MAP2K1, and FGFR1 Are Potential Drug Targets for NSC765598

**Figure 2:**
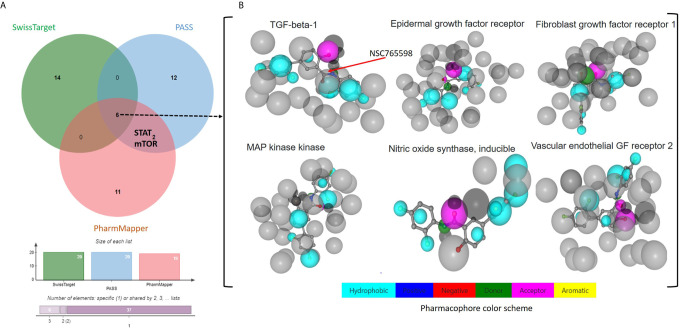
Potential targets for NSC765598. **(A)** Venn diagram showing common targets of NSC765598 as revealed by the SwissTarget, PASS, and PharmMapper algorithms. Six genes including iNOS, TGFB1, vascular endothelial growth factor receptor 2, FGFR, EDFR, and MAPK2 were predicted by the three algorithms (**Figures 3A, B**), while two genes, viz., signal transducer and activator of transcription and serine/threonine-protein kinase mTOR, were predicted to be top ranked targets of NSC765598 by the two algorithms. **(B)** Pharmacophore-based models of the six commonly identified NSC765598 target predictions *via* protein-receptor interactions.

Our PASS analysis of NSC765598 predicted among other activities, anti-inflammatory, antiangiogenic, and anti-proliferative activities, with Pa (probability of being active) values of 0.491, 0.443, and 0.205, respectively and low Pi (probability of being inactive) values of < 0.060, 0.029, and 0.092, respectively. NSC765598 was also predicted to be an antagonist of connective tissue growth factor (Pa = 0.796, Pi = 0.001), platelet-derived growth factor receptor kinase (Pa = 0.695, Pi = 0.007), VEGF (Pa = 0.141, Pi = 0.037), hepatocyte growth factor (HGF) (Pa = 0.132, Pi = 0.018), FGF (Pa = 0.099, Pi = 0.074), MAPK (Pa = 0.137, Pi = 0.005), EGFR (Pa = 0.139, Pi = 0.004), TGFB1 (Pa = 0.150, Pi = 0.051), iNOS (Pa = 0.232, Pi = 0.011), and tumor necrosis factor (TNF)-α (Pa = 0.150, Pi = 0.051) **(** **Supplementary Table 1** **).** Furthermore, NSC765598 target predictions using SwissTarget algorithm partitioned the top 20 targets into three classes: enzymes (20%), kinases (40%), and family A G protein-coupled receptors (40%) **(** **Figure 1B** **).** Coherent with the PASS target predictions, SwissTarget also identified EGFR, TGFB1, FGFR1, iNOS, VEGFR2, MAPK, and serine/threonine-protein kinase mTOR as top druggable targets of NSC765598 (**Supplementary Table 2**). Furthermore, we also used PharmMapper Server, an integrated pharmacophore matching platform with statistical methods to identify potential targets. Interestingly out of 1627 drug targets (459 of which were human protein targets) on PharmMapper, EGFR, TGF-βR1, FGFR1 and −2, VEGFR2, iNOS, MAPK, HGFR, insulin-like growth factor 1 receptor (IGF1R), B-Raf proto-oncogene serine/threonine-protein kinase, serine/threonine-protein kinase Chk1, and cyclin-dependent kinase 5 (CDK5) activator 1 were predicted to be among the top 20 targets for NSC765598. The top 20 predicted targets exhibited three or more total protein-ligand interactions, two or more hydrophobic interactions with a normalized fit score range of 0.7082 to 0.9998, and a z score range of 0.1056 to 0.641082 (**Supplementary Table 3**). Collectively, six genes, viz., *iNOS*, *TGFB1*, *VEGFR2*, *FGFR*, *EGFR*, and *MAPK2* were predicted by the three algorithms (**Figures 2A, B** **)**, while two genes, viz., signal transducer and activator of transcription (*STAT*) and serine/threonine-protein kinase (*mTOR*) were predicted to be NSC765598 top ranked target by 2 algorithms.

### NSC765598 Targets Are Enriched in Cancer-Associated Biological Processes and Signaling Pathways

**Figure 3:**
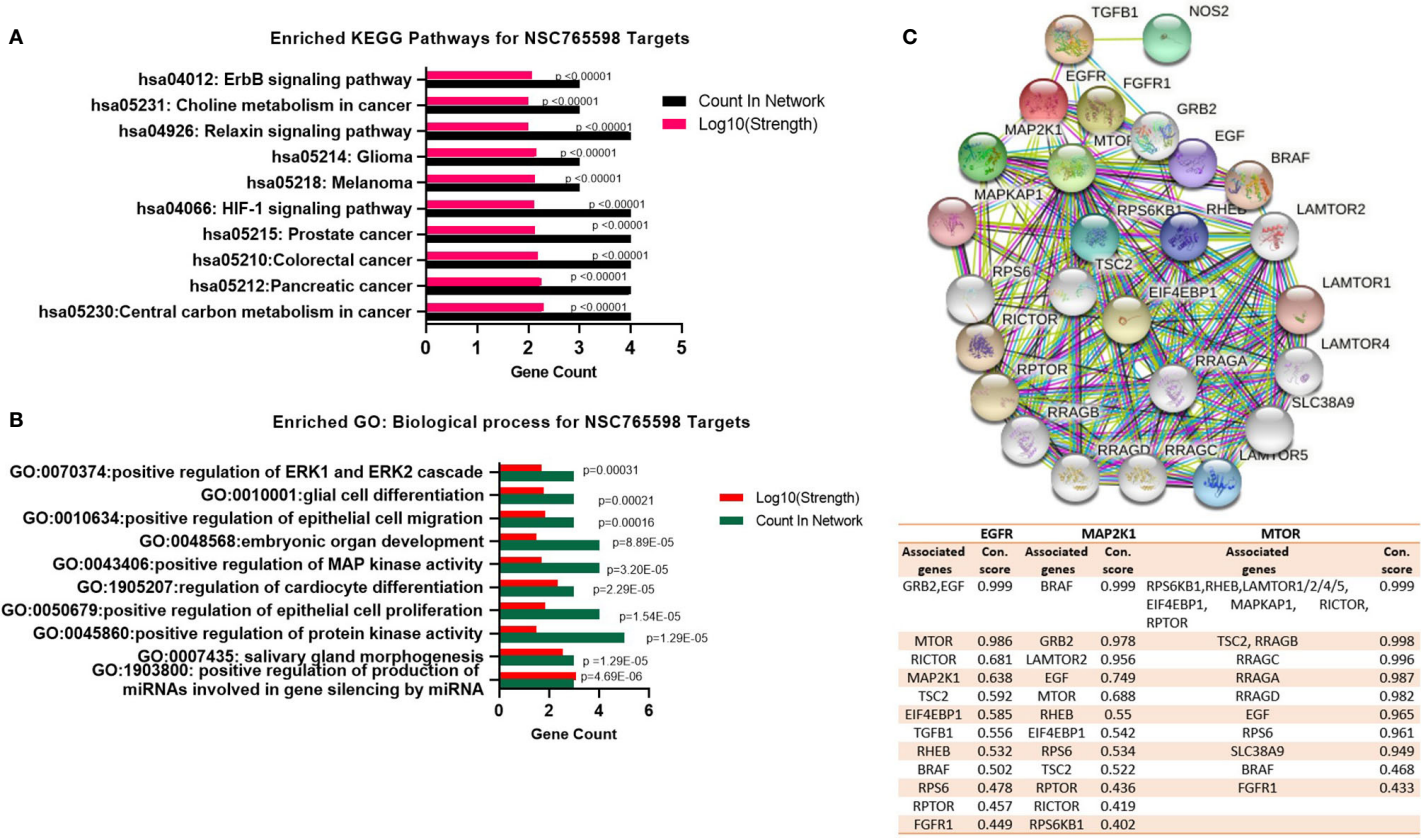
NSC765598 targets are enriched in cancer-associated biological processes and signaling pathways. **(A)** Enriched Kyoto Encyclopedia of Genes and Genomes (KEGG) pathway of NSC765598 targets. Enrichment was considered at p < 10-6, a gene count of ≥ 3, and a strength of ≥ 2. **(B)** Enriched Gene Ontology (GO) of NSC765598 targets. **(C)** Protein-protein interaction (PPI) network of mTOR/EGFR/iNOS/MAP2K1/FGFR/TGFB1. Second-order clustering of NSC765598 gene targets generated 26 nodes, 176 edges, 13.5 average node degrees, an 0.811 average local clustering coefficient, and a PPI enrichment p-value of < 1.0e-16. Hub genes with the highest nodes were mTOR, MAP2K1, and EGFR with 23, 14, and 13 nodes, respectively The accompanying table shows connection scores of nodes with the top three hub genes (mTOR, MAP2K1, and EGFR).

Our second-order clustering of NSC765598 gene targets generated 26 nodes, 176 edges, 13.5 average node degrees, a 0.811 average local clustering coefficient, and a PPI enrichment *p* value of < 1.0e-16. Hub genes with the highest numbers of nodes were mTOR, MAP2K1, and EGFR with 23, 14, and 13 nodes, respectively, and connectivity scores that ranged 0.433 to 0.999 (mTOR), 0.433–0.999 (EGFR), and 0.402 to 0.999 (MAP2K1), while FGFR1 (0.433–0.836), TGFB1 (0.473–0.965), and NOS2 (0.473) had the fewest nodes with four, four, and one, respectively. Genes with the highest interaction scores (con. score = 0.999) with mTOR were *RPS6KB1*, *RHEB*, *LAMTOR1/2/4/5*, *EIF4EBP1*, *MAPKAP1*, *RICTOR*, and *RPTOR*; those with EGFR were *GRB2* and *EGF*; and that with MAP2K1 was *BRAF* (**Figure 3C** ****and** accompanying table**). To gain insights into biological processes and pathways of the selected NSC765598 targets, KEGG pathway and GO enrichment analyses were conducted. As shown in **Figure 3A**, the top 10 KEGG pathways that were significantly enriched (*p* < 10^−5^, gene count of ≥ 3, and strength of ≥ 2) for selected NSC765598 targets included central carbon metabolism in cancer (*p* = 1.516e-9), pancreatic cancer (*p* = 2.720e-9), colorectal cancer (*p* = 4.747e-9), prostate cancer (*p* = 7.739e-9), the hypoxia-inducible factor (HIF)-1 signaling pathway (*p* = 8.756e-9), melanoma (*p* = 8.877e-7), glioma (*p* = 0.000001005), relaxin signaling pathway (*p* = 2.530e-8), choline metabolism in cancer (*p* = 0.000002328) and erbb signaling pathway (*p* = 0.000001468). The top 10 enriched biological processes (**Figure 3B**) were positive regulation of production of miRNAs involved in gene silencing by miRNA (*p* = 4.69E-06), salivary gland morphogenesis (*p* = 1.29E-05), positive regulation of protein kinase activity (*p* = 1.29E-05), positive regulation of epithelial cell proliferation (*p* = 1.54E-05), regulation of cardiocyte differentiation (*p* = 2.29E-05), positive regulation of MAPK activity (*p* = 3.20E-05), embryonic organ development (*p* = 8.89E-05), positive regulation of epithelial cell migration (*p* = 0.00016), glial cell differentiation (*p* = 0.00021), and positive regulation of the extracellular signal-regulated kinase 1 (ERK1) and ERK2 cascade (*p* = 0.00031). Collectively, our observations suggested that the selected NSC765598 targets were enriched in biological processes and pathways associated with growth development and cancer progression.

### EGFR, mTOR, NOS2, TGFB1, MAP2K1, and FGFR1 Are Overexpressed and Are of Prognostic Relevance in Multiple Cancers

**Figure 4:**
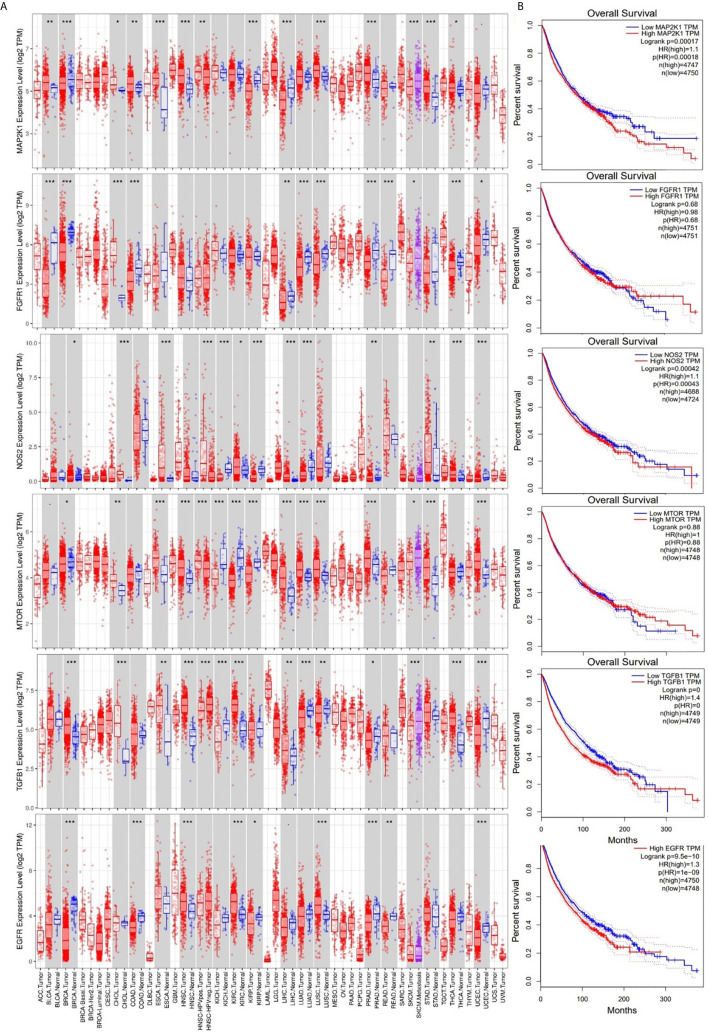
Expression levels and prognostic values of epidermal growth factor receptor (EGFR), mammalian target of rapamycin (mTOR), nitric oxide synthase 2 (NOS2), transforming growth factor-β1 (TGFB1), mitogen-activated protein 2 kinase 1 (MAP2K1), and fibroblast growth factor receptor 1 (FGFR1) in The Cancer Genome Atlas (TCGA) cancer cohorts. **(A)** Box plots showing differential gene expression levels (log2 TPM) of signal transduction and activator of transcription 3 (STAT3)/cyclin-dependent kinase 2 (CDK2)/4/6 between tumor and adjacent normal tissue samples across TCGA database. Blue labels indicate normal tissues, and red labels indicate tumor samples. **(B)** Kaplan-Meier curve of overall survival of cancer cohorts with low and cohorts with high RNA expression levels of EGFR, mTOR, NOS2, TGFB1, MAP2K1, and FGFR1. Higher RNA expression profiles of MAP2K1/NOS2/EGFR were correlated with low overall survival of cohorts. The statistical significance of differentially expressed genes was evaluated using the Wilcoxon test. * *p*<0.05; ** *p*<0.01; *** *p*<0.001.

We analyzed differential expression profiles of EGFR between cancer and matched normal tissues across all TCGA tumors and found that cancer tissues expressed EGFR at significantly (*p* < 0.001) higher levels than did adjacent normal tissues in breast cancer (BRCA), colorectal adenocarcinoma (COAD), HNSC, kidney renal clear cell carcinoma (KIRC), lung squamous cell carcinoma (LUSC), prostate adenocarcinoma (PRAD), rectum adenocarcinoma (READ), and uterine corpus endometrial carcinoma (UCEC); NOS2 at higher levels in cholangiocarcinoma (CHOL), esophageal carcinoma (ESCA), kidney renal papillary cell carcinoma (KIRP), liver hepatocellular carcinoma (LICH), lung adenocarcinoma (LUAD), thyroid cancer (THCH), and UCEC; TGFB1 at higher levels in urothelial bladder carcinoma (BLCA), CHOL, HNSC, kidney chromophobe (KICH), KIRC, LICH, LUAD, LUSC, skin cancer (SKCM), and THCA; mTOR at higher levels in ESCA, HNSC, LICH, LUAD, LUSC, PRAD, stomach adenocarcinoma (STAD), and UECE; MAP2K1 at higher levels in BRCA, ESCA, HNSC, KIRP, LICH, LUSC, PRAD, STAD, LUAD, THCA, and UECE; and FGFR1 at higher levels in BLCA, BRCA, CHOL, COAD, LUAD, LUSC, PRAD, and THCA than adjacent normal tissues (**Figure 4A**). However, cancer tissues expressed mTOR at significantly (*p* < 0.001) lower levels than adjacent normal tissues in KICH, KIRC, and KIRP; FGFR1 at lower levels in READ and THCA; and NOS2 at lower levels in KICH than adjacent normal tissues. In addition, we explored the prognostic relevance of EGFR, mTOR, NOS2, TGFB1, mTOR, and FGFR1. First, we grouped the 9736 TCGA cancer cohorts into high or low RNA expressions of EGFR, mTOR, NOS2, TGFB1, mTOR, and FGFR1 and compared the survival of cohorts from the two groups. Results showed that cohorts with higher mRNA expressions of EGFR, MAP2K1, and NOS2 exhibited shorter overall survival (OS) than the cohort with lower expression profiles. However, there was no significant correlation (*p* > 0.05) with OS for TGFB1, mTOR, or FGFR1 expressions (**Figure 4B**). Higher EGFR expression was also found to be associated with poor DFS of the cohorts, whereas mTOR, NOS2, TGFB1, MAP2K1, and FGFR1 expressions were not associated with DFS of the cohorts (**Supplementary Figure 1**).

**Figure 5:**
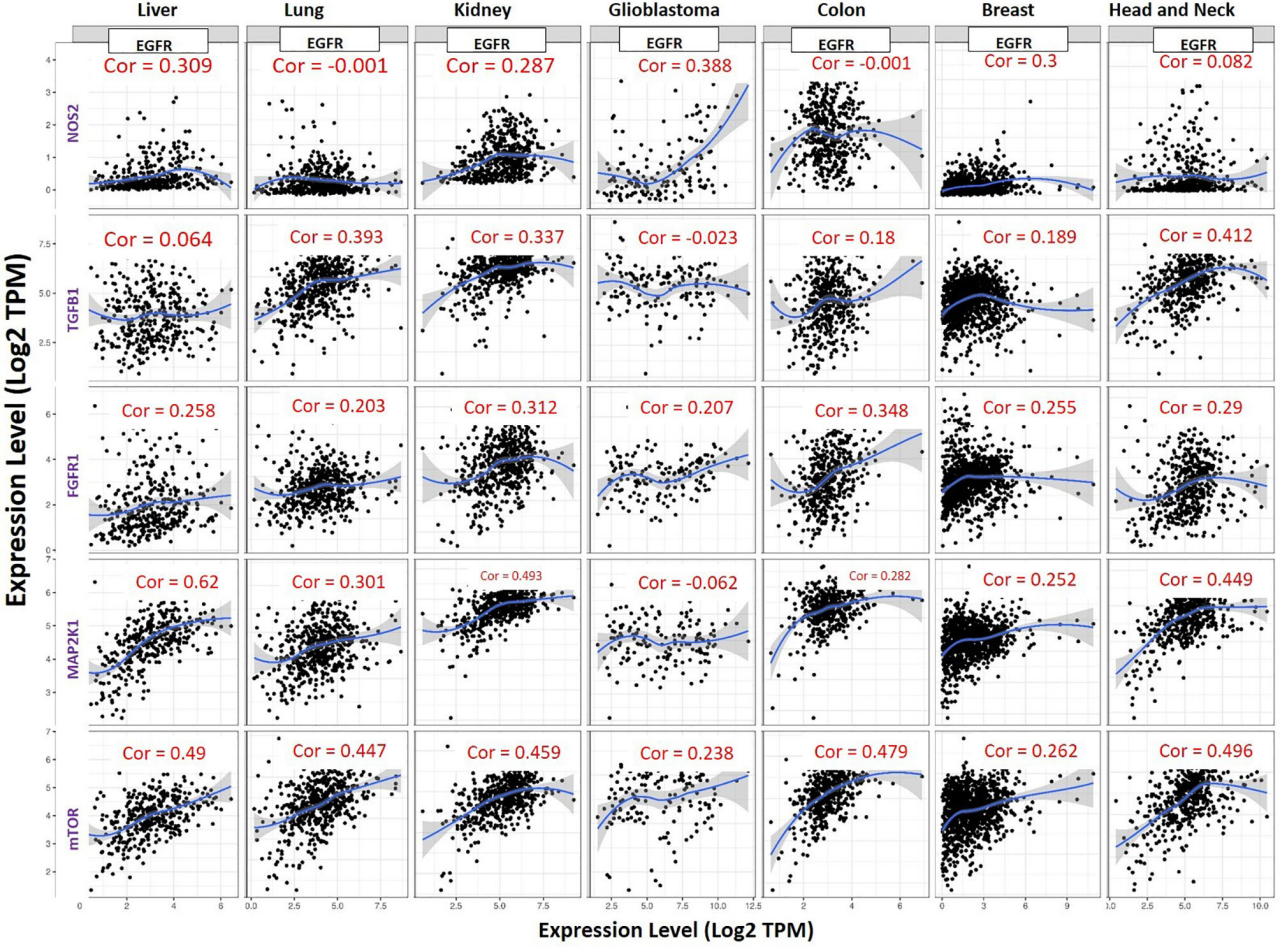
Expression scatterplots of EGFR correlations with NOS2, TGFB1, MAP2K1, mTOR and FGFR1 in multiple cancer types. The strength of correlations between the genes is reflected by the purity-adjusted partial spearman’s rho value, where a value of *r*= 1 means a perfect positive correlation and a value of *r*= −1 means a perfect negative correlation.

Our correlation analysis revealed that EGFR expression was strongly correlated with expressions of mTOR (r= 0.262–0.496) and FGFR1 (r= 0.203–0.348) in liver, lung, kidney, glioblastoma, colon, head and neck, and breast cancers. MAP2K1 expression was strongly correlated with expressions of EGFR (r= 0.252–0.62) in liver, lung, kidney, colon, head and neck, and breast cancers but negatively correlated in glioblastoma (r = −0.062). EGFR expression was strongly correlated with expressions of NOS2 (r= 0.287–0.388) in liver cancer, kidney cancer and, breast cancer, poorly correlated in head and neck cancer (r=0.082) but show no association (r = −0.001) in colon and lung cancer (**Figure 5**).

### Genetic alterations of EGFR Are Associated With Poor Prognoses of Cancer Patients

**Figure 6:**
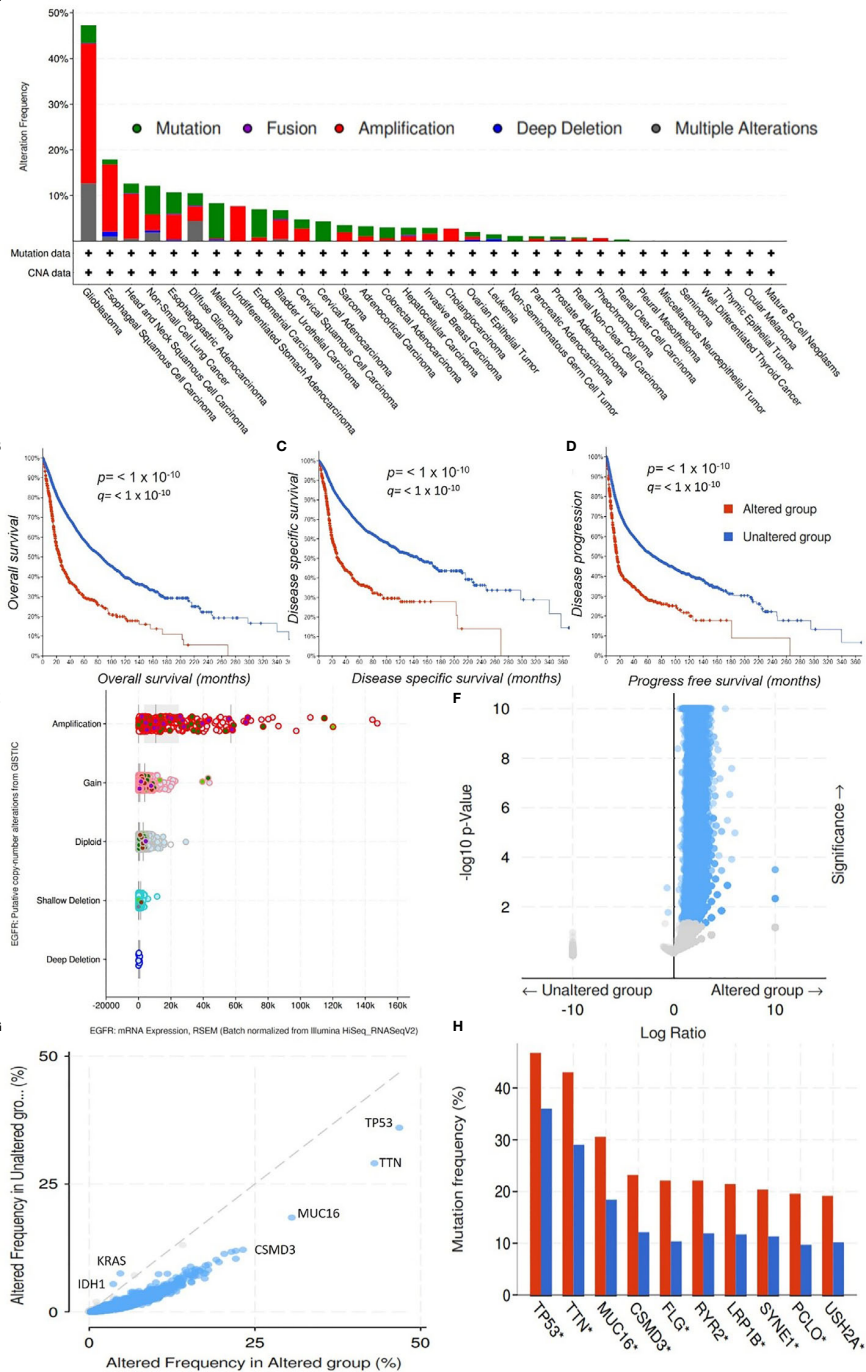
Genomic alterations in the epidermal growth factor receptor (EGFR) are associated with poor prognoses of cancer cohorts. **(A)** Bar graph showing alteration frequencies of the EGFR in cohorts of different cancer types. The most common alteration in the *EGFR* gene was amplification. Kaplan-Meir survival curve of **(B)** overall survival, **(C)** disease-specific survival, and **(D)** disease-progression survival of cancer cohorts with altered *EGFR* and unaltered *EGFR* genes. Cohorts with altered *EGFR* had low overall survival, disease-specific survival, and disease-progression survival. *p* < 10^−10.^ **(E)**. Bar showing the EGFR putative copy number alterations and messenger RNA expressions. **(F)** Heat map showing *p* values and significance levels of other gene mutations associated with cohorts with altered *EGFR* and cohorts with unaltered *EGFR*. **(G)** Line graph showing frequencies of altered genes in cancer cohorts with altered *EGFR* and cohorts with unaltered *EGFR*. **(H)** Bar graph showing gene mutations that were significantly enriched in both *EGFR*-altered and *EGFR*-unaltered cohorts.

We explored the cancer genomic dataset from the cBioPortal to analyze genomic alterations in EGFR, iNOS, mTOR, FGFR, TGFR, and MAP2K1 of 10,953 cancer patients (**10,967** samples) from different cancer types. We found that of the 10,967 cancer cohorts representing 33 cancer types, genetic alterations in EGFR occurred in 821 (7%) cohorts of 26 cancer types (**Figure 6A**). EGFR mutations occurred most frequently in glioblastomas (47.3%), esophageal squamous cell carcinoma (17.89%), head and neck squamous cell carcinoma (12.62%), NSCLC (12.16%), esophagogastric adenocarcinomas (10.7%), and diffuse gliomas (10.53%), while pleural mesothelioma, seminoma, well-differentiated thyroid cancer, thymic epithelial tumor, ocular melanoma, and mature B cell neoplasm cohorts were devoid of EGFR mutations (**Figure 6A**). The most common alterations of *EGFR* gene were amplification and gene gain, while gene deletions were the least common alterations in *EGFR* genes (**Figures 6A, E** **)**. These alterations in *EGFR* genes were associated with low OS (*p* < 10^−10^), disease-specific survival (*p* < 10^−10^), and disease-progression survival (*p* < 10^−10^) in those cohorts (**Figures 6B** **–D**). We also determine the gene alteration co-occurrence frequencies with respect to EGFR-altered and EGFR-unaltered cohorts and found a number of other gene mutations that co-occurred with alteration of EGFR (**Figures 6F, G** **)**. The top ten gene mutations that were significantly (*p* value= 1.07 × 10^−23^ to 4.79 × 10^−21^ and q value 2.07 × 10^−19^ to 9.32 × 10^−18^) enriched in EGFR-altered cohorts were *RICTOR*, *UGGT1*, *ATRNL1*, *AHNAK2*, *RIMS2*, *DSP*, *STXBP5L*, *DUOX2*, *PKD1L1*, and *MYH3* (**Figure 6F**, **Table 2**), while only two genes, viz., *KRAS* (*p* = 11.7 × 10^−03^ and q = 2.22 × 10^−03^) and *IDH1* (*p* = 0.017 and q = 0.0194) were enriched in unaltered EGFR cohorts (**Figure 6G**, **Table 2**). However, 10 genes, including *TP53* (46.78% and 36.01%), *TTN* (43.03% and 29.05%), *MUC16* (30.56% and 18.43%), *CSMD3* (29.19% and 12.15%), *FLG* (22.12% and 10.38%), *RYR2* (22.12% and 11.92%), *LRP1B* (21.45% and 11.73%), *SYNE1* (20.38% and 11.34%), *PCLO* (19.57% and 9.71%), and *USH2A* (19.17% and 10.18%) had higher mutation frequencies in both EGFR-altered and EGFR-unaltered cohorts, respectively (**Figure 6H**). However, only 6% (662/10953), 4% (478/10967), 3% (346/10967), 1% (141/10967), and 1% (133/10967) of cohorts had *FGFR*, *mTOR*, *iNOS*, *TGFB1*, and *MAP2K1* gene alterations **(** **Supplementary Figure 2** **)** and were not associated (*p* > 0.05) with poor survival or prognosis **(** **Supplementary Figures 3–7** **)**.

**Table 2:** Enriched genes in EGFR altered and Unaltered cohorts across the cbioportal database.

Gene	Cytoband	Altered group	Unaltered group	Log Ratio	p-Value	q-Value	Enriched
RICTOR	5p13.1	66 (8.85%)	164 (1.69%)	2.39	1.07 × 10^−23^	2.07 × 10^−19^	Altered group
UGGT1	2q14.3	59 (7.91%)	130 (1.34%)	2.56	2.40 × 10^−23^	2.33 × 10^−19^	Altered group
ATRNL1	10q25.3	77 (10.32%)	242 (2.50%)	2.05	2.61 × 10^−22^	1.45 × 10^−18^	Altered group
AHNAK2	14q32.33	130(17.43%)	631 (6.51%)	1.42	4.12 × 10^−22^	1.45 × 10^−18^	Altered group
RIMS2	8q22.3	98 (13.14%)	386 (3.98%)	1.72	4.35 × 10^−22^	1.45 × 10^−18^	Altered group
DSP	6p24.3	91 (12.20%)	337 (3.48%)	1.81	4.48 × 10^−22^	1.45 × 10^−18^	Altered group
STXBP5L	3q13.33	78 (10.46%)	261 (2.69%)	1.96	3.31 × 10^−21^	8.64 × 10^−18^	Altered group
DUOX2	15q21.1	68 (9.12%)	199 (2.05%)	2.15	3.55 × 10^−21^	8.64 × 10^−18^	Altered group
PKD1L1	7p12.3	92 (12.33%)	358 (3.69%)	1.74	4.64 × 10^−21^1	9.32 × 10^−18^	Altered group
MYH3	17p13.1	73 (9.79%)	231 (2.38%)	2.04	4.79 × 10^−21^1	9.32 × 10^−18^	Altered group
IDH1	2q34	27 (3.62%)	526 (5.43%)	−0.58	0.017	0.0194	Unaltered group
KRAS	12p12.1	35 (4.69%)	728 (7.51%)	−0.68	1.7 × 10^−3^	2.22 × 10^−03^	Unaltered group

### Molecular Docking Revealed Unique Interactions of NSC765598 With EGFR, mTOR, NOS2, TGFB1, MAP2K1, and FGFR1

In order to evaluate the strength and nature of interactions between NSC765598 and the selected mapped pharmacophores, NSC765598 was docked into the active sites of EGFR, mTOR, NOS2, TGFB1, MAP2K1, and FGFR1.

#### Molecular Docking of NSC765598 With mTOR

**Figure 7:**
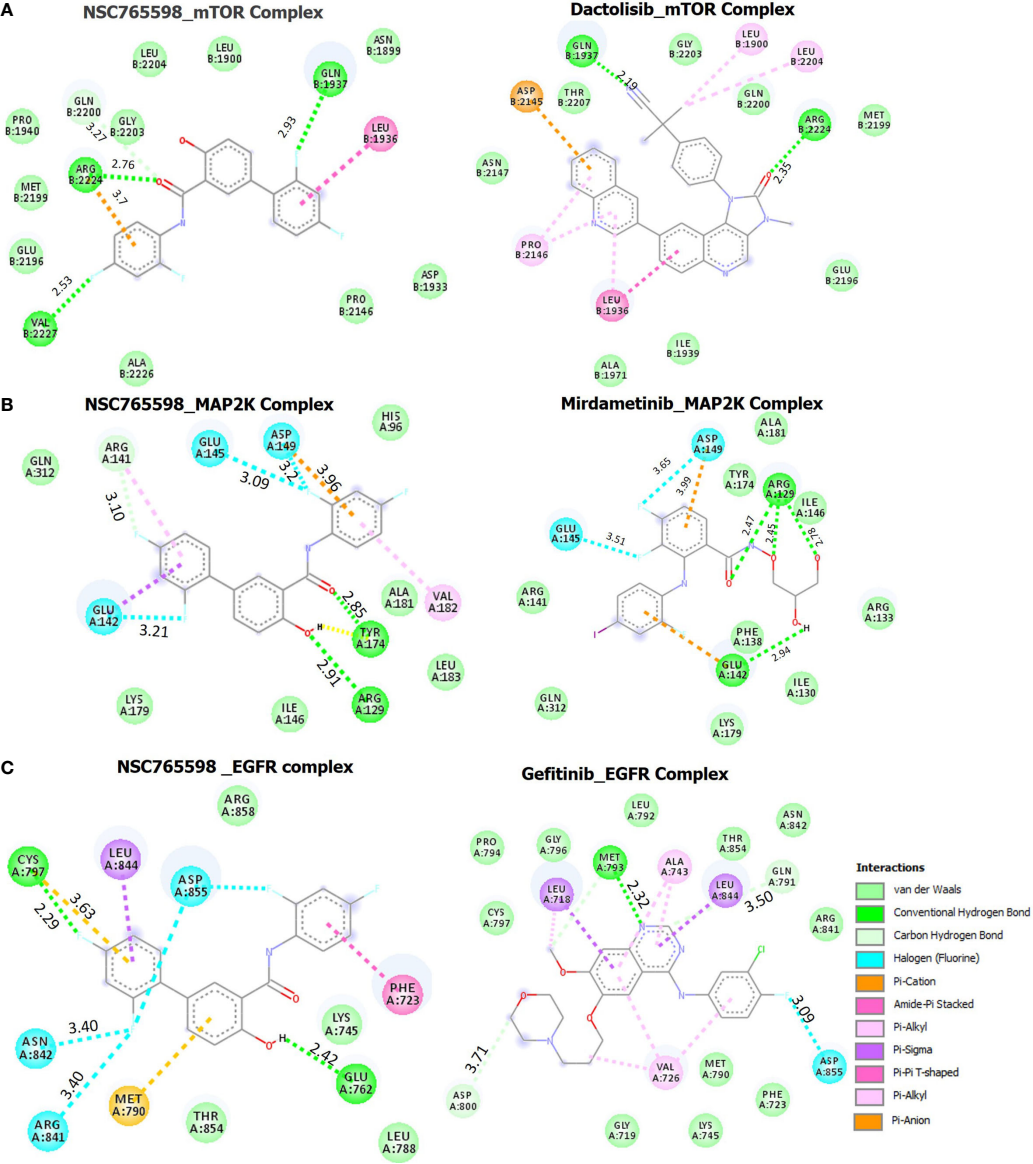
Docking profiles of mTOR/MAP2K/EGFR with NSC765598 and standard inhibitors. The two dimensional (2D) representations of ligand–receptor complexes, showing the interacting amino acid residues and the type of interactions, occurring between the ligands and **(A)** mTOR **(B)** MAP2K and **(C)** EGFR.

NSC765598 demonstrated a –8.8 kcal/mol binding affinity for mTOR. The NSC765598- mTOR complex was bonded by conventional H-bonds with GLN1937, VAL2227, and ARG2224, C-H bonding with GLN2200, halogen bonding with GLN1937 and VAL2227, and multiple π-interactions. NSC765598 also forms 4 hydrophobic contacts with LEU1936, GLN1937, and GLN2200 residues of mTOR. Stabilization of NSC765598– mTOR complex was also supported by the Van der Waal forces between the ligand and ASP1933, PRO21146, ALA2226, GLU2196, MET2199, PRO1940, GLY2203, LEU2204, LEU1900, and ASN1899 residues of the receptor binding pocket **(** **Figure 7A** **)**. However, a standard mTOR inhibitor, dactolisib exhibited a higher affinity for mTOR (−9.2 kcal/mol) than NSC765598 **(** **Table 3** **)**.

**Table 3:** NSC765598 and standard drug comparative docking profile against mTOR/MAP2K/EGFR.

	mTOR				MAP2K				EGFR			
	NSC765598	DIS (Ӑ)	Dactolisib	DIS (Ӑ)	NSC765598	DIS (Ӑ)	Mirdamatinib	DIS (Ӑ)	NSC765598	DIS(Ӑ)	Gefitinib	DIS (Ӑ)
ΔG=(Kcal/mol)	−8.8		−9.2		−7.6		−6.5		−11.0		−6.8	
Conventional	ARG2224	2.76	GLN1937	2.19	ARG129	2.91	ARG129	2.78	GLU762	2.42	MET793	2.32
H-bond	VAL2227	2.53	ARG2224	2.35	TYR174	2.85	GLU142	2.94	CYS792	2.29		
	GLN1937	2.83										
C-H bond	GLN2200	3.27			ARG141	3.1					ASP800	3.71
											GLN791	3.50
Halogen bond	GLN1937	3.36			ASP149	3.20	ASP149	3.65	ASN842	3.24	ASP855	3.09
	Val2227	3.30			GLU145	3.09	GLU145	3.51	ARG841	3.40		
					GLU142	3.21			ASP855	3.52		
π -anion			ASP2145	4.82	ASP149	3.96	ASP149	3.99				
							GLU142	4.59				
π -cation	ARG2224	4.27										
π –sulfur		3.70							MET790 CYS797	5.43 3.63		
π -alkyl			PRO2146		ARG141						ALA743	
			LEU1900								VAL726	
			LEU2204									
π -π stacked									PHE723			
Amide-π stacked	LEU1936		LEU1936									
					GLU142				LEU844	5.49	LEU844	
π-sigma											LEU718	
Van der waal forces	ASP1933,		ALA1971		GLN312		VAL182		ARG858		GLY719	
	PRO21146,		ILE1939		LYS179		ALA181		LYS745		LYS745,	
	ALA2226,		GLU2196		ILE146		TYR174		LEU788		PHE723,	
	GLU2196,		MET2199		ALA181		ILE146		THR854		MET790,	
	MET2199,		GLN2200		LEU183		ARG133				ARG841,	
	PRO1940		GLY2203		HIS96		PHE138				ASN842,	
	GLY2203		THR2207				LYS149				THR854	
	LEU2204		ASN2147				GLN312				LEU792,	
	LEU1900						ARG141				GLY796,	
	ASN1899										PRO794, CYS797	
Hydrophobic Interactions	sLEU1936	3.89	LEU1900	3.31	ARG141	3.99	LEU50	3.84	PHE723	3.74	LEU718	3.77
	GLN1937	3.73	PRO2146	3.63	GLU142	3.70	VAL58	3.67	PHE723	3.66	LEU718	3.70
	GLN1937	3.58	GLU2196	3.97	GLU142	3.64	MET121	4.0	PHE723	3.88	LEU718	3.90
	GLN2200	3.61	GLN2200	3.77	GLU145	3.84	LEU173	3.51	LEU844	3.42	VAL726	3.59
							THR186	3.64	LEU844	3.64	VAL726	3.59

π –sulfur: π-electron cloud between the Aromatic rings of ligands and lone pair of electron cloud of sulfur atom in the receptors; π -π stacked: π-electron cloud between the Aromatic rings, π -π T-shaped: T shaped π-electron cloud between the Aromatic rings, π –alkyl; π-electron cloud between the Aromatic ring of ligand and alkyl group of ligand.

#### Molecular Docking of NSC765598 With MAP2K

Molecular docking studies revealed that NSC765598 docked well into the MAP2K binding cavity with more robust interactions, stronger binding affinity (−7.6 kcal/mol), and shorter interaction distances (2.85–3.96 Å) than the interaction observed between MAP2K and a standard inhibitor, mirdamatinib (−6.5 kcal/mol, 2.78–4.59 Ă). There NSC765598-MAP2K complex was bonded by conventional H-bonds with Arg129 and Tyr174, a carbon-hydrogen bond with Arg141, halogen bonding with Glu145, Glu142, and Asp149, and multiple π-interactions, including π-anion interaction with Asp149, π-sigma interaction with Glu142, and π-alkylation with Arg141. NSC765598 also forms 4 hydrophobic contacts with ARG141, GLU142 and GLU145 residue of MAP2K. Stabilization of the NSC765598-MAP2K complex was also supported by Van der Waal forces between the ligand and the amino acid residues (Gen312, Lys179, Ile146, Leu183, Ala181, and His96) in the receptor binding pocket **(** **Figure 7B**, **Table 3** **).**

#### Molecular Docking of NSC765598 With EGFR

Molecular docking of NSC765598 with EGFR revealed more robust interactions and higher binding affinity (−11 kcal/mol) to EGFR than do gefitinib (−6.8 kcal/mol) a standard EGFR inhibitor. The NSC765598-EGFR complex was bonded by strong H-bond interactions with GLU762 and CYS797, halogen bond interactions with ASN842, ARG841, and ASP855, and multiple π-interactions, including π-π interaction with PHE723, π-alkyl interaction with LEU844, and π-sulfur interactions with MET790 and CYS797 **(** **Figure 7C** **)**. In terms of interaction distances between the ligand atoms and receptor atoms, the hydrogen bond interactions of GLU762 (2.42 Å) and CYS797 (2.29 Å) were the shortest, while π-interactions of LEU844 (5.49 Å), CYS797 (3.63 Å), and MET790 (5.43 Å) had longer distance interactions within the NSC765598-EGFR complex. NSC765598 also forms 5 hydrophobic contacts with PHE723 and LEU844 residues of EGFR. Stabilization of the NSC765598-EGFR complex was also supported by Van der Waal forces between the ligand and ARG858, LYS745, LEU788, and THR854 in receptor binding pockets **(** **Figure 7C**, **Table 3** **).**

#### Molecular Docking of NSC765598 With FGFR1

**Figure 8:**
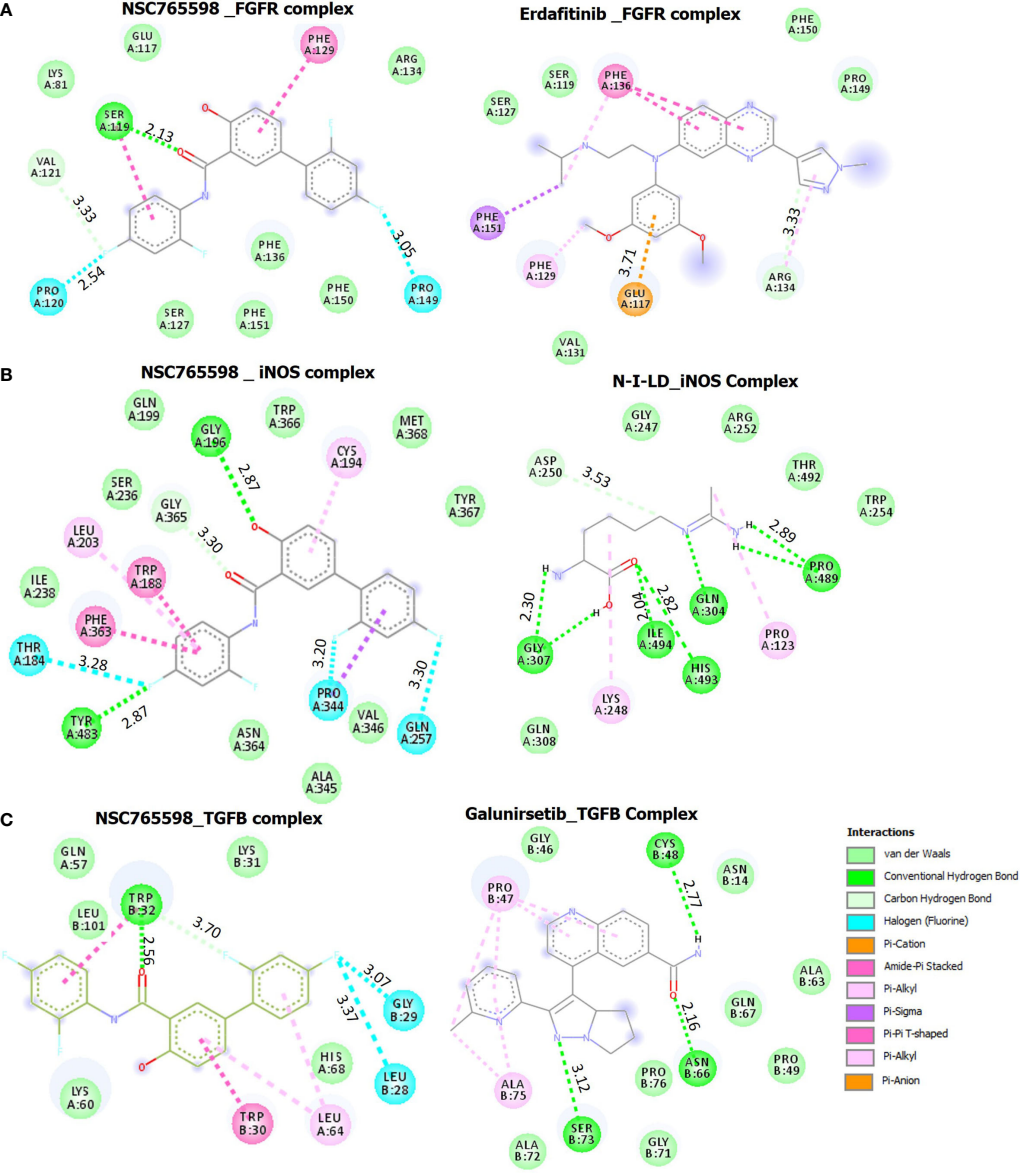
Docking profiles of iNOS/FGFR/TGFB with NSC765598 and standard inhibitors. The two-dimensional (2D) representations of ligand–receptor complexes, showing the interacting amino acid residues and the type of interactions, occurring between the ligands and **(A)** iNOS **(B)** FGFR and **(C)** TGFB.

NSC765598 docked well with the binding pocket of FGFR1 with a binding affinity of −7.3 kcal/mol stronger than the interactions between FGFR and Erdafitinib (−5.7 Kcal/mol). The NSC765598-FGFR1 complex was stabilized by two H-bonds with SER119 (2.13 Å) and VAL121 (3.33 Å) as opposed to only single H-bond interaction (ARG134) between FGFR_Erdafitinib. In addition, halogen interactions (PRO120, 2.54 Ă and PRO149, 3.05 Ă), π-π stacking with the aromatic ring of PHE129, and Amide-Pi stacking with SER119 were also observed between the NSC765598_FGFR1 complex. NSC765598-FGFR1 complex was also supported by several Van der Waal forces created around the backbone of the ligand with respective amino acid residues (LYS81, GLU117, ARG134, PHE136, PHE151, PHE150, and SER127) of the receptor binding pocket **(** **Figure 8A**, **Table 4** **)**.

**Table 4:** NSC765598 and standard drug comparative docking profile against iNOS/FDFR/TGFB.

	iNOS			FGFR					TGFB1			
	NSC765598	DIS (Ă)	N-I-LD	DIS (Ă)	NSC765598	DIS (Ă)	Erdafitinib	DIS (Ă)	NSC765598	DIS (Ă)	Galunisertib	DIS (Ă)
ΔG=(Kcal/mol)	– 11.0		– 5.3		−7.3		−5.7		−7.2		−7.1	
Conventional H-bond	TYR483	2.65	GLY307	2.30	SER119	2.13	–		TRP32	2.56	CYS48	2.77
	GLY196		ILE494								ASN66	2.16
	GLY363	2.87	GLN304	2.04							SER73	3.12
		3.30	PRO489	2.26								
			HIS493	2.89 2.82								
C-H bond			ASP250	3.53	VAL121	3.33	ARG134	3.33	TRP32	3.70		
Halogen bond	GLN257	3.30			PRO120	2.54			GLY29	3.07		
	PRO344	3.20			PRO149							
	THR184	3.28				3.05						
π -anion							GLU117	3.71	LEU28	3.37		
π -alkyl	CYS194,		LYS248				PHE129		LEU64		PRO47	
	LEU203,		PRO123				ARG134				ALA75	
	PRO344											
π -π stacked	TRP188,				PHE129				TRP32			
	PHE363								TRP30			
Amide-π stacked					SER119							
π -π T-shaped							PHE136					
π-sigma							PHE151					
Van der waal forces	ILE238,		GLY247		GLU117,		SER127,		LEU101,		GLY46, ASN14,	
	SER236,		ARG252		ARG134,		SER119,PHE150,		GLN57,		ALA63, GLN67,	
	GLN199,		THR492		PHE136,		PRO149,		LYS31,LYS60,		PRO49, PRO76,	
	TRP366,		TRP254		PHE151,		VAL131		HIS68		GLY711, ALA72	
	MET368,		GLN308		PHE150,							
	TYR367,				LYS81,							
	ALA345,				SER127							
	VAL346,											
	ASN364											
Hydrophobic interaction	TRP188	3.83	ASP250	3.91	PHE129	3.58	PHE129		TRP32	3.44	PRO47	3.52
	TRP188	3.66			PHE136		PHE136		TRP32	3.97		
	PRO344	3.81			PHE136	3.78	PHE151		LEU64	3.81		
	PHE363	3.63				3.50						

π-sulfur, π-electron cloud between the Aromatic rings of ligands and lone pair of electron cloud of sulfur atom in the receptors; π-π stacked: π-electron cloud between the Aromatic rings, π-π T-shaped: T shaped π-electron cloud between the Aromatic rings, π-alkyl; π-electron cloud between the aromatic ring of ligand and alkyl group of the receptor, N-I-LD, N-Iminoethyl-l-lysine dihydrochloride.

#### Molecular Docking of NSC765598 With iNOS

Molecular docking of NSC765598 with iNOS revealed a binding affinity of −11.0 kcal/mol. NSC765598 interacts with iNOS by three H-bonds with TYR483, GLY196, and GLY363, and halogen interactions with GLN257, PRO344, and THR184. Binding interaction proximities in the NSC765598-iNOS complex ranged 2.65–3.28 Å; the shortest distance interactions were conventional H-bonds with TYR483 (2.65 Å) and GLY196 (2.87 Å). The NSC765598-iNOS complex was also supported by π-interactions; π-π stacking with aromatic rings of TRP188 and PHE363, and π-alkyl interactions with CYS194, LEU203, and PRO344. The interaction was also stabilized by Van der Waal forces created on the backbone with the following amino acids: ILE238, SER236, GLN199, TRP366, MET368, TYR367, ALA345, VAL346, and ASN364 **(** **Figure 8B**, **Table 4** **)**. Furthermore, NSC765598 interaction with iNOS is more robust and with higher affinity than the interactions between iNOS and N-Iminoethyl-L-lysine dihydrochloride (−5.3 Kcal/mol), a standard iNOS inhibitor **(** **Table 4** **).** In addition, NSC765598 forms 4 hydrophobic contact with TRP188, PRO344, and PHE363 residue of iNOS as compared to 1 hydrophobic contact in the iNOS_N-Iminoethyl-L-lysine dihydrochloride complex

#### Molecular Docking of NSC765598 With TGF-β1

NSC765598 docked well to the binding cavity of TGF-β1 with an affinity of −7.2 kcal/mol comparable with the affinity that galunisertib (a standard TGF-β1 inhibitor) has for TGF-β1 (−7.1 kcal/mol). NSC765598 binds with TGF-β1 by single H-bond (TRY32) as against three (3) H-bonds (CYS48, ASN66, SER73) between galunisertib_TGF-β1 complex. In addition to the halogen interactions (GLY29 and LEU28), π-stacking with the aromatic ring of TRP30, and π-alkyl interaction with LEU64 residues of TGF-β1, NSC765598-TGF-β1 complex was also supported by Van der Waal forces created between the ligand backbone and amino acid residues (LEU101, GLN57, LYS31, and HIS68) of TGF-β1 binding pocket **(** **Figure 8C**, **Table 4** **).** In addition, NSC765598 forms 3 hydrophobic contact with TRP32 and LEU64 residues of TGFB as compared to 1 hydrophobic contact (PRO47) in the TGF-β1_ galunisertib complex.

#### Anti-Proliferative Activities of NSC765598 Against NCI60 Panels of Human Tumor Cell Lines

**Figure 9:**
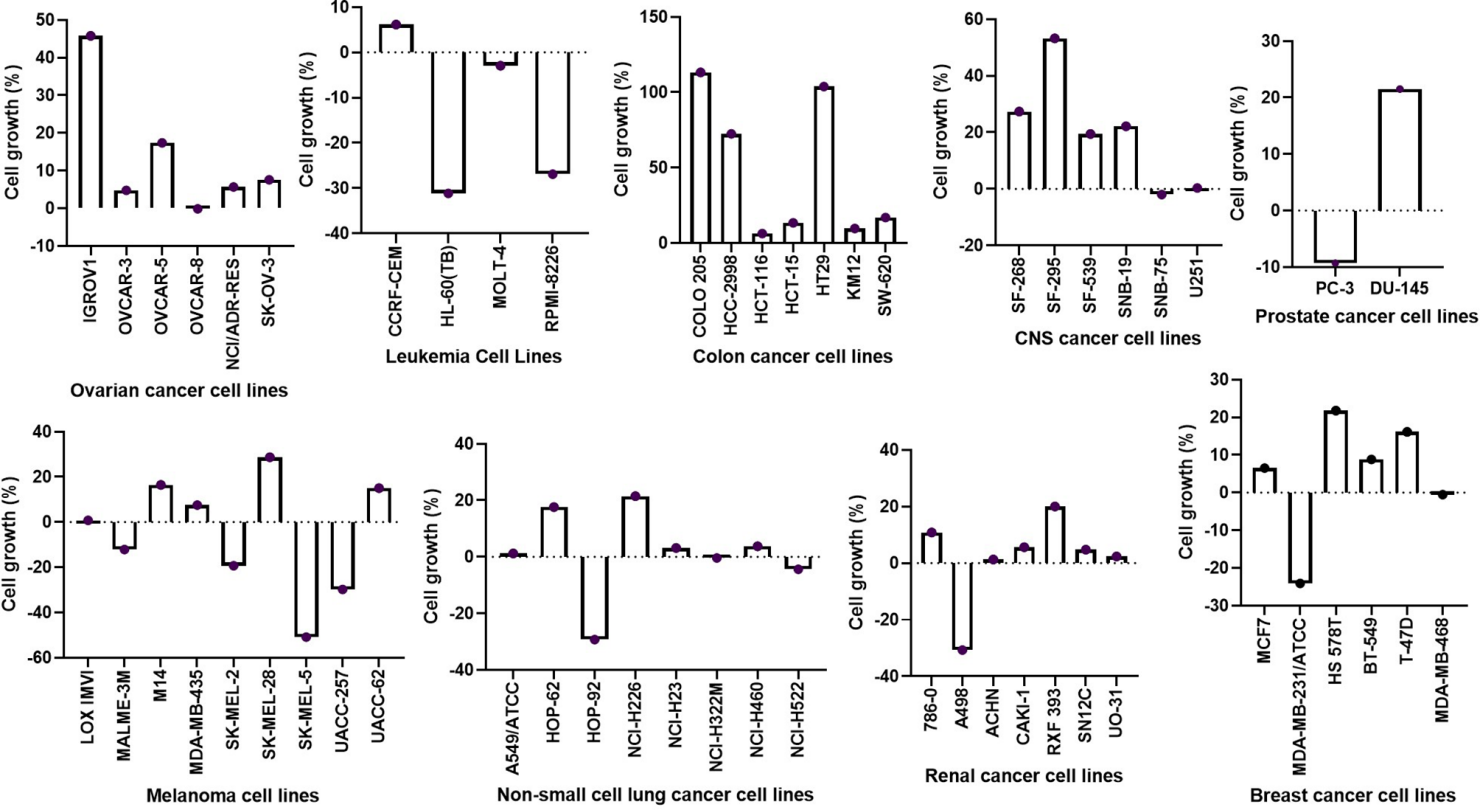
Inhibitory activities of NSC765598 against panels of 60 human cancer cell lines. Each cell line was treated with a single dose of 10 μM of NSC765598 for 48 h. The bar points above point zero on the y-axis indicate the mean percentage of cell growth relative to cells without drug treatment, while bar points below point zero indicate the percentage of cell death.

The percent growth inhibition (GI) caused by single-dose testing revealed that NSC765598 inhibited the growth of all of the NCI60 cell line panels of breast, prostate, renal, ovarian, colon, melanoma, CNS, leukemia, and non-small cell lung cancers. Single-dose (10 μM) treatment with NSC765598 exhibited more than 50% growth inhibition of all the NCI60 cell line panels except for two colon cancer cell lines (HT29 and COLO 205) which were completely insensitive to NSC765598 treatment (**Figure 9**). Furthermore, single-dose treatment with NSC765598 also demonstrated cytotoxic activities against leukemia cell lines of HL-60 (cell growth (CG) = −31.31%) and RPMI-8226 (CG = −26.88%), NSCLC cell lines of HOP-92 (CG = −29.23%) and NSC-H522 (CG = −4.38%), melanoma cell lines of MALME-3M (GI = −12.11%), SK-MEL-2 (CG = −19.27%), SK-MEL-5 (CG = −50.91%), and UACC-257 (CG = −29.80%), renal cell line of A498 (CG = −30.67%), prostate cell line of PC-3 (CG = −9.28%), and breast cancer cell line of MDA-MB-231/ATCC (CG = −24.07%). However, no cytotoxic activities were detected against the ovarian, colon, or CNS cancer cell lines (**Figure 9**). The primary single-dose results clearly indicated the anticancer activities of NSC765598 against different kinds of human cancer cell lines, and thus it is worthy of further evaluation for dose-dependent activities.

### NSC765598 Exhibited Selective Cytotoxic Preferences for NSCLC, Leukemia, Melanoma, and Renal Cancer Cell Lines

**Figure 10:**
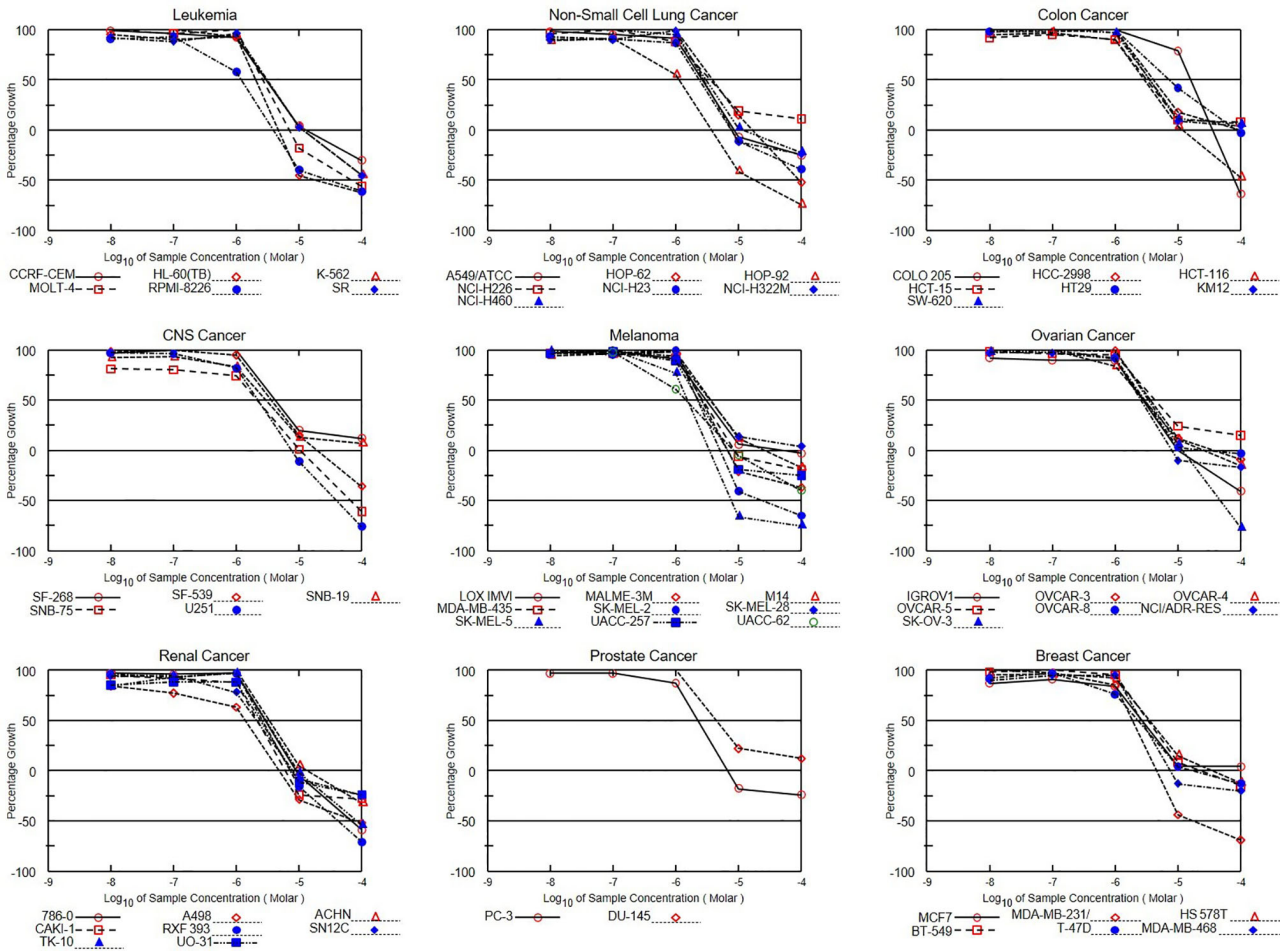
Dose-response curves of NCI60 human cancer cell lines to NSC765598 treatment. Growth percentage value of 100 on the y-axis represents the growth of untreated cells, the 0 value represents no net growth, while −100 represents complete death of cells.

**Figure 11:**
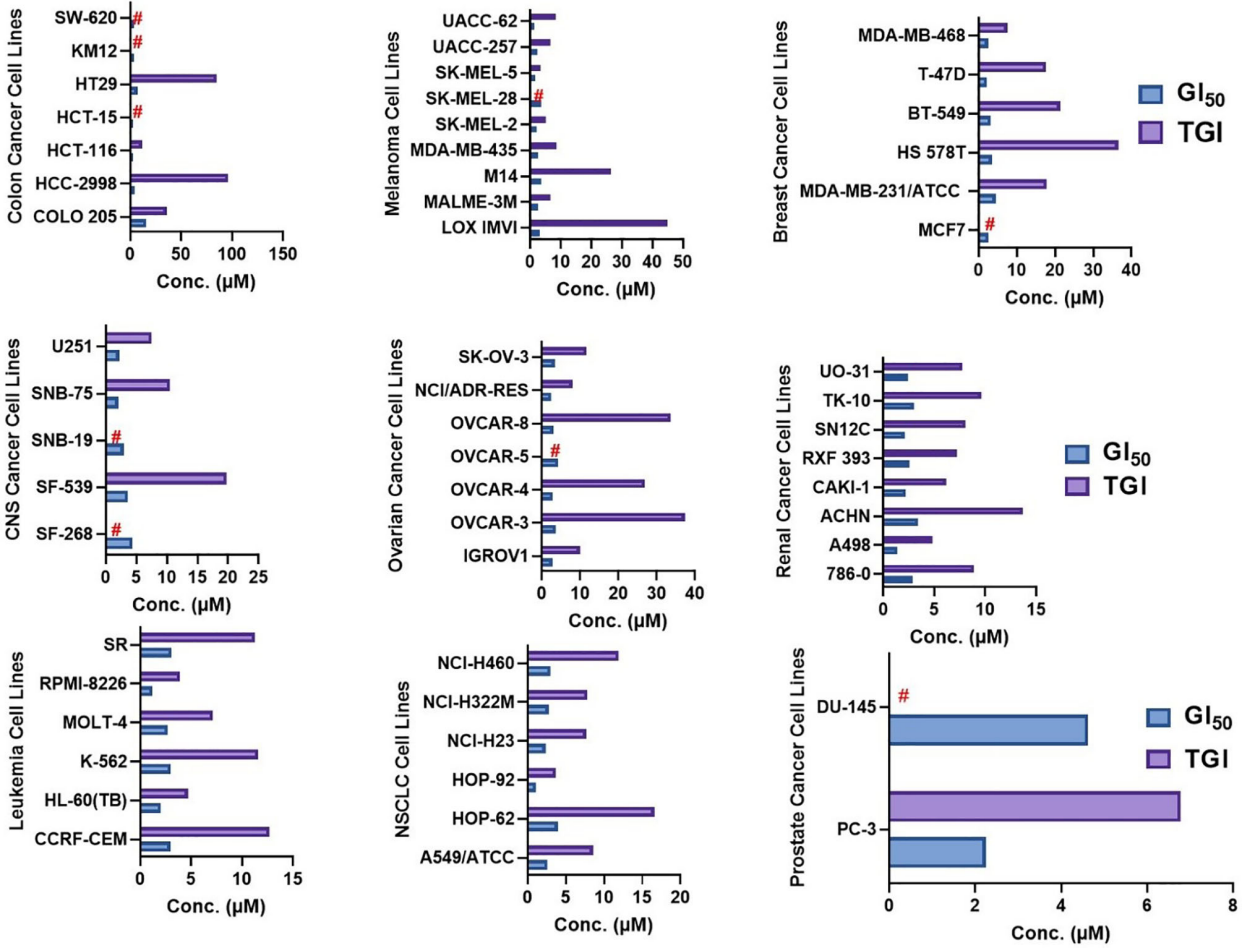
GI_50_ (50% inhibition of the cancer cell growth) and TGI (100% growth inhibition of the cancer cells) concentrations of NSC765598 against NCI60 panels of human cancer cell lines.

In the five-dose assay screening, NSC765598 demonstrated selective dose-dependent cytotoxic effects against the panel of NCI60 human tumor cell lines **(** **Figure 10** **).** The GI_50_ values of NSC765598 (concentration of NSC765598 that causes 50% inhibition of cell growth) against individual cells of the NCI60 panels of human cell lines tested were lower than 5.0 µM, except for COLO 205, a colon cancer cell line with a GI_50_ of 16.0 µM (**Figure 11**). However, among the nine types of cancer involved in this assay, panels of NSCLC cell lines were the most responsive to growth inhibition by NSC765598 treatment (GI_50_ = 1.12–3.95 µM) followed by leukemia cell lines (GI_50_ = 1.20–3.10 µM), melanoma cell lines (GI_50_ = 1.45–3.59 µM), and renal cell lines (GI_50_ = 1.38–3.40 µM). Furthermore, NSC765598, displayed the least TGI (concentration causing 100% growth inhibition of cancer cells) against six panels of NSCLC cell lines (TGI =3.72–16.60 μM), six panels of leukemia cell lines (TGI = 3.90–12.70 μM), and eight panels of renal cancer cell lines (TGI = 4.84–13.70 μM), while the highest TGI (11.80 to > 100 μM) and perhaps the lowest activity were recorded for the seven panels of colon cancer cell lines. Furthermore, as revealed by the LC_50_ (concentration causing 50% lethality of cancer cells), cytotoxic activities of NSC765598 were more pronounced against NSCLC cell lines of HOP-92 (LC_50_ = 18.90 μM) and HOP-62 (LC_50_ = 92.0 μM), leukemia cell lines of HL-60 (LC_50_ = 20.30 μM), RPMI-8226 (LC_50_ = 29.50 μM), and MOLT-4 (LC_50_ = 71.10 μM), melanoma cell lines of SK-MEL-5 (LC_50 =_ 7.68 μM) and SK-MEL-2 (LC_50_ = 23.50 μM), brain cancer cell lines of U251 (LC_50_ = 40.0 μM) and SNB-75 (LC_50_ = 65.70 μM), and renal cancer cell lines of RXF 393 (LC_50_ = 41.60 μM), 786-0 (LC_50_ = 67.0 μM), A498 (LC_50_ = 78.70 μM), and TK-10 (LC_50_ = 84.20 μM), while the two panels of prostate cancer cell lines (PC-3 and DU-145) were less responsive to the cytotoxic effects of NSC765598 (LC_50_ > 100 μM). However, contrary to the primary one-dose cell growth percent inhibition assay, the colon cancer cell line, COLO 205 was found to be the most sensitive to five-dose testing of NSC765598 than the other colon cancer cell lines, displaying total growth inhibition and 50% cytotoxic response at NSC765598 concentrations of 35.80 and 80.30 μM, respectively **(** **Figure 11**, **Table 5** **).**

**Table 5:** LC_50_ (50% lethality of the cancer cells) concentrations of NSC765598 against NCI60 panels of human cancer cell lines.

	Leukemia		NSCLC		Breast Cancer		CNS Cancer	
	Cell lines	LC_50_	Cell lines	LC_50_	Cell lines	LC_50_	Cell Lines	LC_50_
1	CCRF-CEM	> 100	A549/ATCC	> 100	MCF7	> 100	SF-268	> 100
2	HL-60(TB)	20.30	HOP-62	92.00	MDA-MB-231/ATCC	0.00	SF-539	> 100
3	K-562	> 100	HOP-92	18.90	HS 578T	> 100	SNB-19	> 100
4	MOLT-4	71.10	NCI-H23	> 100	BT-549	> 100	SNB-75	65.70
5	RPMI-8226	29.50	NCI-H322M	> 100	T-47D	> 100	U251	40.00
6	SR	> 100	NCI-H460	> 100	MDA-MB-468	> 100		
	**Melanoma**		**Ovarian Cancer**		**Renal Cancer**		**Colon Cancer**	
	**Cell lines**	**LC_50_**	**Cell lines**	**LC_50_**	**Cell lines**	**LC_50_**	**Cell lines**	**LC_50_**
1	LOX IMVI	> 100	IGROV1	> 100	786-0	67.00	COLO205	80.30
2	MALME-3M	> 100	OVCAR-3	> 100	A498	78.70	HCC-2998	> 100
3	M14	> 100	OVCAR-4	> 100	ACHN	> 100	HCT-116	> 100
4	MDA-MB-435	> 100	OVCAR-5	> 100	CAKI-1	> 100	HCT-15	> 100
5	SK-MEL-2	23.50	OVCAR-8	> 100	RXF 393	41.60	HT29	> 100
6	SK-MEL-28	> 100	NCI/ADR-RES	> 100	SN12C	> 100	KM12	> 100
7	SK-MEL-5	7.68	SK-OV-3	47.00	TK-10	84.20	SW-620	> 100
8	UACC-257	> 100			UO-31	> 100		
9	UACC-62	> 100						
	**Prostate Cancer**							
	**Cell lines**	**LC_50_**						
1	PC-3	> 100						
2	DU-145	> 100						

### NSC765598 Share a Similar Antitumor Fingerprint With NCI Synthetic Compounds and Standard Agents

DTP-COMPARE analysis indicated that NSC765598 share similar antitumor fingerprints with a number of NCI standard agents. The p-value and cell counts of the top 15 NCI correlated standard agents are shown in **Table 6**. All NCI synthetic compounds with NSC765598 similar antitumor fingerprints are small molecules (MW; 277.3–461.9 g/mol). The identity, p-value, cell counts, and molecular weight of the top 15 NCI synthetic compounds are also shown in **Table 6**.

**Table 6:** NCI synthetic compounds and standard anticancer agent sharing similar anti-cancer fingerprints and mechanistic correlation with NSC765598.

Rank	p	CCLC	Target NSC	Small Molecules	MW: (g/mol)	Correlation	CCLC	Standard Anti-cancer
1	0.89	57	758440	Niclosamide (USAN)	327.12	0.42	56	4-ipomeanol
2	0.84	55	50681	N-(2-ethoxyphenyl)-3-hydroxynaphthalene-2-carboxamide	307.3	0.39	55	Bryostatin 1
3	0.77	54	772889	MK-2048	461.9	0.35	44	Flavoneacetic Acid Ester
4	0.76	57	757391	Dichlorophene	269.1	0.34	55	Tamoxifen
5	0.76	57	37202	Celcot RN	313.3	0.33	56	Rapamycin
6	0.72	55	37188	Azotol OT	277.3	0.33	56	Flavoneacetic Acid
7	0.7	57	37608	Naphthol AS-AN	308.2	0.32	47	Morpholino-ADR
8	0.68	56	777205	3-(4-chloro-2-fluorophenyl)-2H-chromen-2-one	274.6	0.32	44	Spirohydantoin Mustard
9	0.67	57	50686	Naphtanilide TR	311.8	0.31	56	SR2555 (Nitroimidazole)
10	0.67	48	13235	Dibromosalicil	400.0	0.31	53	Piperazine Alkylator
11	0.64	57	766722	3-(1H-benzimidazol-2-yl)-6-hexyl-2-(2-methylphenyl)iminochromen-7-ol	451.6	0.3	57	Rapamycin
12	0.62	57	81947	Dibromsalan (USAN)	3.71.0	0.29	56	Merbarone
13	0.62	56	760057	Nitazoxanide (USAN)	307.2	0.29	46	O6-Methylguanine
14	0.62	52	705084	4-Butanediyldihydroguaiaretic	330.4	0.29	56	DTIC
15	0.61	57	12969	N-(3-Chlorophenyl)-3-hydroxynaphthalene-2-carboxamide	297.7	0.29	56	Fluorodopan

P, Pearson’s correlation coefficient; CCLC, common cell lines count; MW, molecular weight.

## Discussion

The application of multi-omics approaches has aided our understanding of carcinogenesis and the development of therapeutic strategies (44). Identifying drug targets for small molecules and their molecular mechanisms play important roles in drug design, discovery, and development (45). Drug targets can be identified *via* genetic interactions, biochemical methods, and computational studies (46). In this study, our collective computational approach identified EGFR/mTOR/NOS2/TGFB1/FGFR1/MAP2K1 as potential targets for NSC765598. The PPI network revealed that these gene targets interact strongly with other oncogenic proteins and were mainly enriched in pathways and processes associated with multiple cancers. In accordance with our observations, previous studies had identified these gene signatures as cancer-associated genes. For instance, Murugan et al. (47) reported that mTOR promotes cancer growth, drug resistance, and metastasis, while McCubrey et al. (48) reported that the EGFR/mTOR signaling pathways play prominent roles in malignant transformation, therapeutic resistance, cancer stemness, metastasis, and inhibition of apoptosis. MAP2K was implicated in hypoxia-mediated angiogenesis, cancer progression, metastasis, and drug resistance (49). Increasing evidence also implicated EGFR signaling pathways in promoting cancer growth and metastasis (50, 51). Taking together, these results strongly suggest the oncogenic roles of EGFR/mTOR/NOS2/TGFB1/FGFR1/MAP2K1 signatures in multiple cancers. Hence, we explored public databases to investigate the full prognostic relevance of these target genes in multiple cancers.

Comparative gene expression profiling of cancer and adjacent normal tissues facilitates an understanding of disease etiologies and enhances the design of appropriate therapeutic interventions (16). Our differential expression analysis revealed that mRNAs of EGFR, mTOR, NOS2, TGFB1, FGFR1, and MAP2K1 were overexpressed in multiple cancer types compared to corresponding adjacent healthy cohorts, while the survival analysis revealed that cohorts with higher expressions of EGFR, MAP2K1, and NOS2 exhibited shorter OS than cohorts with low expression profiles. Collectively, these data suggested that EGFR/mTOR/NOS2/TGFB1/FGFR1/MAP2K1 play important roles in the progression of tumors and serve as reliable biomarkers of poor prognosis

Using the cBioPortal, we also studied frequencies of genetic alterations of these signatures and their prognostic values in cancer patients. We found that genetic alterations in EGFR occurred in 7% of cancer cohorts, most frequently in GBM, ESCC, HNSCC, and NSCLC, and were associated with poor prognoses and survival of patients. Dong et al. (52) evaluated the alteration status of the top three genes identified as prognosticators of renal cell carcinoma and respectively found only 2%, 2%, and 1.7% genetic alterations in the *FN1*, *COL1A2*, and *COL3A1* genes. Chen et al. (53) studied genetic alterations of 20 genes and found alteration frequencies ranging 0.8% to 3% for 19 individual genes, while only one gene (*NBN*) had an alteration frequency of 9%. Therefore, percentages of patients with genetic alterations in *EGFR* (7%), *FGFR* (6%), *mTOR* (4%), and *iNOS* (3%) as reported in this study were on the high side and thus strengthen our earlier observations that these signatures are very important in cancer progression and can thus serve as attractive targets worthy of further exploration.

Nevertheless, it is becoming increasingly evident that the initiation and progression of cancer cannot be ascribed to a single genetic mutation (54). Therefore, we evaluated the co-occurrence of genetically altered *EGFR* with other genetic mutations in the cancer cohorts, and we found its co-occurrence with genetically altered *RICTOR*, *UGGT1*, *ATRNL1*, *AHNAK2*, *RIMS2*, *DSP*, *STXBP5L*, *DUOX2*, *PKD1L1*, and *MYH3*. Alterations in these genes were found to be positively enriched in patients with altered *EGFR*, while alterations in *KRAS* and *IDH1* were only enriched in patients without genetically altered *EGFR*. Intriguingly, we also observed that the most common genetic alterations in the *EGFR* gene were amplifications and gene gains, supporting the role of EGFR as an oncoprotein. These findings are also coherent with our earlier observations on the differential expression of EGFR mRNA in cancer cohorts compared to healthy cohorts. However, contrary to our earlier observations on the poor survival rates of cohorts with high mRNA expressions of EGFR, mTOR, FGFR iNOS, TGFB1, and MAP2K1, only genetic alterations of *EGFR*, but not those of *mTOR*, *iNOS*, *TGFB1*, or *MAP2K1*, were associated with poor prognoses and survival of patients.

Lung cancer is the most commonly diagnosed (often diagnosed in advanced stages) and the leading cause of global cancer mortality due to limited therapeutic success (3, 55). It is therefore plausible to say that genetic alterations in *EGFR* are a major contributor to this dilemma. In line with our observations, Ding et al (56). reported that the EGFR pathway is the most altered pathway in lung adenocarcinomas (26%). Gower et al (57). identified that alterations in *EGFR* are major oncogenic drivers of acquired resistance and predict poor survival in NSCLC, while Saadeh et al (58). reported high correlations of *EGFR* mutations with poor therapeutic responses and survival of glioblastoma patients. Although, treatments with EGFR tyrosine kinase inhibitors (TKIs) showed initial therapeutic responses, acquired resistance to 1st-, 2nd-, and recently approved 3rd-generation TKIs (osimertinib) owing to EGFR mutations has been well reported (59, 60). Taken together, patients with high EGFR mRNA expression are more likely to harbor genetically altered *EGFR* and experience worse prognoses. This necessitates alternative treatment strategies; hence, we evaluated NSC765598 for that purpose.

In order to facilitate the identification of potent anticancer small molecules from synthetic libraries, the National Cancer Institute (NCI) established the NCI60 cell panel screen to examine the activities of small molecules on cancer cell viability (61). Hence, we explored this avenue to evaluate the anticancer properties of NSC765598 against the NCI60 panel of cell lines. Interestingly we found that NSC765598 exhibited antiproliferative effects on all panels of breast cancer, prostate cancer, renal cancer, ovarian cancer, melanoma, CNS cancer, leukemia, and NSCLC cells with GI_50_ values of < 5 µM, but it showed lower anti-proliferative activities against colon cancer cell lines. Among the nine types of cancer involved in this assay, NSC765598 had some cytotoxic preference for NSCLC, leukemia, melanoma, and renal cancer cell lines. Interestingly, NSC765598 also exhibited activity against a drug-resistant cell line, NCI/ADR-RES (GI_50_ = 2.54 µM; TGI = 8.00 μM). However, the least amounts of cell lethality were found against panels of colon, breast, ovarian, prostate, and CNS cancer cell lines (TGI > 20 µM in most cases and LC_50_ >100 µM in almost all cases), suggesting that NSC765598 is not generally toxic to growing cell lines, but displays some degree of specificity to NSCLC, renal cancer, leukemia, and melanomas. The NCI60 cell lines used in this study have been well characterized for proteins, genes, microRNA expressions, mutations, and DNA methylation  (62–64). Mutations in the EGFR TK domain have been reported in NSCLC and have been associated with response to gefitinib and erlotinib (65, 66). In addition, among the NCI60 cell lines, mutations in the EGFR gene have been identified in* *the leukemia cell line (RPMI-8226) and melanoma cell line (SK-MEL-28) and were found to be associated with the resistant of these cell lines to 12 tyrosine kinase inhibitors (TKI) including the erlotinib (67, 68). It is, therefore, noteworthy that NSC765598 demonstrated high activity on these cells line (**Figure 11**, **Table 5**).

COMPARE analysis indicated that NSC765598 shares a similar antitumor fingerprint with a number of NCI standard agents and a very strong correlation with NCI synthetic compounds (p-value 0.61~0.89). Importantly, the top most correlated synthetic compounds, niclosamide (p-value 0.89) is a known anticancer agent, which have been mechanistically reported to exhibit anticancer activities *via* suppression of Wnt/β-catenin, mTORC1, EGF, STAT3, NF-κB, and Notch signaling pathways (69, 70), while nitazoxanide (p-value 0.62) exhibit it anticancer activities *via* suppression of MAPK and mTOR pathways (71). The strong correlation of these compounds with the NSC765598 anticancer fingerprint strongly suggests a similar mechanism of action. Hence, it is very likely that anticancer activities demonstrated by NSC765598 could be attributed to suppression of EGF, MAPK, and mTOR signaling pathways, which is in line with the in silico target prediction and molecular docking. Collectively, this study has provided *in vitro* and in silico evidence for anticancer activities of NSC765598 and inhibition of EGFR, mTOR, NOS2, TGFB1, MAP2K1, and FGFR1 as the most probable mechanism of action. Hence we validated this hypothesis using molecular docking of ligand-protein interactions.

Molecular docking of a drug candidate with target proteins is very useful for identifying the strength of an association between a ligand and receptor and ultimately gives a prediction of the activity of the small molecule (18). We conducted a molecular docking study to elucidate likely binding affinities and binding interactions of NSC765598 with selected targets. We found that NSC765598 docked well into the binding cavity of EGFR, mTOR, NOS2, TGFB1, FGFR1, and MAP2K1 with binding affinities ranging −7.2 to −11.0 kcal/mol. Hydrogen bonds together with other non-covalent interactions, such as hydrophobic and ionic interactions and van der Waals forces, play important roles in stabilizing protein-ligand interactions (72). Interestingly, interactions of all targets with NSC765598 predominantly involved conventional hydrogen bonds, fluorine bonds, hydrophobic contacts, van der Waal forces, and a variety of π-interactions including π-π stacking, and π-anion and π-cation interactions, which consequently led to high binding affinities and strong ligand-NSC765598 complexes, and likely compromised the expression integrity of the proteins.

Among the targets, EGFR and iNOS demonstrated unique interactions with a higher binding affinity of −11 kcal/mol compared to other targets, thereby suggesting higher promising inhibitory effects of NSC765598 against EGFR and iNOS. The high binding affinities and unique stability of NSC765598 in binding pockets of iNOS and EGFR were chiefly attributed to three conventional H-bonds with TYR483, GLY196, and GLY363 of iNOS and two conventional H-bonds with GLU762 and CYS797 of EGFR; halogen interactions with GLN257, PRO344, and THR184 of iNOS, and ASN842, ARG841, and ASP855 of EGFR; and multiple hydrophobic and Pi interactions (π-stacking, π-alkyl, and π-sulfur). The large numbers of π-interactions, which mostly involve charge transfer, helped NSC765598 intercalate in binding cavities of the receptors. Furthermore, higher Van der Waal forces created on the NSC765598 backbone with the respective amino acids of ILE238, SER236, GLN199, TRP366, MET368, TYR367, ALA345, VAL346, and ASN364 in the binding pocket of iNOS; and ARG858, LYS745, LEU788, and THR854 in the binding pocket of EGFR created strong cohesive environments, thereby stabilizing the complexes formed (73).

The critical role of amino acids in docking has been well demonstrated (74). Alkylation of proteins is dependent on the biochemical properties of target amino acids to create nucleophilic sites (75). Therefore, the specific alkylation of the LEU844 residue of EGFR and alkylation of the three CYS194, LEU203, and PRO344 residues of iNOS by NSC765598 could mediate the modification of substrate binding sites and subsequently induce alkylation-dependent inhibition of target proteins (76). Collectively, the strong interactions observed between NSC765598 and EGFR/iNOS may compromise their activity/expression abilities and consequently affect the survival of cells that depend on these proteins for their vital activities. These findings, therefore, suggest that the anticancer activities demonstrated by NSC765598 could be attributed more to its dual inhibition of iNOS and EGFR. Specifically, the docking results corroborated our observations of NSCLC cell lines, which were observed to demonstrate higher responses to NSC765598 (GI_50_ = 1.12–3.95 µM; TGI = 3.72–16.60 μM). Therefore, stronger NSC765598 interactions with EGFR could be implicated in the higher activity of NSC765598 on NSCLC panels. Interestingly, NSC765598 demonstrated more robust interactions and higher binding affinities for MAP2K, EGFR, iNOS, and FGFR1 than does their respective standard drugs; mirdametinib, gefitinib, N-Iminoethyl-l-lysine dihydrochloride, and erdafitinib. However, it shows, lower binding affinity for mTOR than dactolisib, and its comparable to galunisertib in its affinity for TGFB1. Currently, there are no iNOS inhibitors approved for human use because promising results in animal studies have not translated to humans (77). Therefore, the results of the present study indicate that NSC765598 could serve as a novel iNOS inhibitor worthy of further preclinical and clinical developments.

Hydrophobic interactions play important role in the formation of ligand-receptor complexes. The number of contacts between hydrophobic atoms reflects the extent of the hydrophobic interactions between a ligand and receptor complex. NSC765598 forms several hydrophobic contacts with amino acid residues of the mTOR/EGFR/MAP2K/iNOS/FGFR/TGFB1 **(** **Tables 3** and **4** **).** These hydrophobic interactions are key players in stabilizing the energetically-favored ligands, in an open conformational environment of the proteins (78). It’s noteworthy that NSC765598 forms higher hydrophobic contacts with the receptors than do some of the standard inhibitors used for comparison in this study **(** **Table 4** **)**. This higher number of hydrophobic contacts in the active core of the drug -target interface would further increase the biological activity of the drug lead. Collectively, this study suggested that NSC765598 has a potential for multi-target inhibition of EGFR/iNOS/mTOR/TGFB1/FGFR/MAP2K1 and could serve as a lead compound for developing new therapeutics for cancer treatment. Preclinical toxicity study is an important aspect of drug discovery and developments (79). Our computational study of toxicity indicated that NSC765598 displayed LD_50_ at high concentration and, thus may be safely used for acute administration, while the drug-likeness studies indicated that it met acceptable criteria of being used as a drug for therapeutic applications. Further *in vitro* and *in vivo* studies in tumor-bearing mice are ongoing to evaluate the full therapeutic efficacy of this novel small molecule.

## Conclusion

The current study identified mTOR/EGFR/iNOS/MAP2K1/FGFR/TGFB1 as potential targets of NSC765598 with higher binding preferences for EGFR, iNOS, and mTOR. NSC765598 displayed anti-proliferative activities and selective cytotoxic preferences for NSCLC, leukemia, melanoma, and renal cancer cell lines. In addition, NSC765598 displayed favorable properties as a drug lead compound and thus could be considered a novel small molecule with potential for multi-target inhibition of EGFR/iNOS/mTOR/TGFB1/FGFR/MAP2K1 and could serve as a lead compound for developing new therapeutics for cancer treatment.

## Data Availability Statement

The original contributions presented in the study are included in the article/**Supplementary Material**. Further inquiries can be directed to the corresponding authors.

## Author Contributions

BL wrote the manuscript. C-YL, NM, and HK helped with data collection and analyses. H-SH and MS synthesized and provided NSC765598. H-SH and AW designed and oversaw the study. All authors contributed to the article and approved the submitted version.

## Funding

H-SH is funded by the Ministry of Science and Technology (MOST109-2113-M-038-003). C-YL is funded by the Ministry of Science and Technology (MOST 109-2314-B-038-108).

## Conflict of Interest

The authors declare that the research was conducted in the absence of any commercial or financial relationships that could be construed as a potential conflict of interest.
