# Supplementary material for: mTOR/EGFR/iNOS/MAP2K1/FGFR/TGFB1 Are Druggable Candidates for N-(2,4-Difluorophenyl)-2′,4′-Difluoro-4-Hydroxybiphenyl-3-Carboxamide (NSC765598), With Consequent Anticancer Implications
Source: Front Oncol. 2021 Mar 26;11:656738. doi: 10.3389/fonc.2021.656738 (PMC8034425; doi:10.3389/fonc.2021.656738)
Supplement: Supplementary file 1 [file DataSheet_1.docx]

Supplementary DATA

**Supplementary Table 1:** PASS prediction of the biological activities of NSC765598

| **Pa** | **P1** | **Activities** |
| --- | --- | --- |
| 0.796 | 0.001 | CTGF expression inhibitor |
| 0.695 | 0.007 | Platelet derived growth factor receptor kinase inhibitor |
| 0.606 | 0.073 | Membrane integrity agonist |
| 0.550 | 0.101 | Nootropic |
| 0.498 | 0.021 | Transcription factor STAT inhibitor |
| 0.491 | 0.060 | Antiinflammatory |
| 0.443 | 0.029 | Angiogenesis inhibitor |
| 0.270 | 0.066 | Proto-oncogene tyrosine-protein kinase Fgr inhibitor |
| 0.260 | 0.199 | CDK9/cyclin T1 and CDK2/cyclin A inhibitor |
| 0.232 | 0.011 | inducible Nitric oxide synthase antagonist |
| 0.205 | 0.092 | Proliferative diseases treatment |
| 0.150 | 0.051 | Transforming growth factor beta-1 antagonist |
| 0.141 | 0.037 | Vascular endothelial growth factor antagonist |
| 0.139 | 0.004 | Epidermal growth factor receptor antagonist |
| 0.137 | 0.005 | Mitogen-activated protein kinase antagonist |
| 0.132 | 0.018 | Hepatocyte growth factor antagonist |
| 0.102 | 0.039 | Protein kinase (PKA, PKC, AKT, GRK, AGC-related, RSK, DBF2, SGK) inhibitor |
| 0.099 | 0.074 | Fibroblast growth factor antagonist |
| 0.073 | 0.046 | Breast cancer-resistant protein inhibitor |

Pa; probability, Pi; probability to be inactive

**Supplementary Table** 2; Swiss Target Prediction of NSC765598 targets

| **Target** | **Common name** | **Uniprot**  **ID** | **ChEMBL**  **ID** | **Target**  **Class** |
| --- | --- | --- | --- | --- |
| Vascular endothelial growth factor receptor 2 | VEGFR2 | P35968 | CHEMBL279 | Kinase |
| Dopamine D1 receptor | DRD1 | P21728 | CHEMBL2056 | AG-PCR |
| Serotonin 6 (5-HT6) receptor | HTR6 | P50406 | CHEMBL3371 | AG-PCR |
| Serotonin 2b (5-HT2b) receptor | HTR2B | P41595 | CHEMBL1833 | AG-PCR |
| Interleukin-8 receptor B | CXCR2 | P25025 | CHEMBL2434 | AG-PCR |
| MAP kinase-interacting serine/threonine-protein kinase | MKNK1 | Q9BUB5 | CHEMBL4718 | Kinase |
| Adenosine A1 receptor | ADORA1 | P30542 | CHEMBL226 | AG-PCR |
| Adenosine A2a receptor | ADORA2A | P29274 | CHEMBL251 | AG-PCR |
| Testis-specific androgen-binding protein | SHBG | P04278 | CHEMBL3305 | Secreted protein |
| Nitric-oxide synthase, brain | NOS1 | P29475 | CHEMBL3568 | Enzyme |
| Nitric oxide synthase, inducible | NOS2 | P35228 | CHEMBL4481 | Enzyme |
| Nitric-oxide synthase, endothelial | NOS3 | P29474 | CHEMBL4803 | Enzyme |
| Serine/threonine-protein kinase mTOR | MTOR | P42345 | CHEMBL2842 | Kinase |
| Phospholipase A2 group IIA | PLA2G2A | P14555 | CHEMBL3474 | Enzyme |
| Fibroblast growth factor receptor 1 | FGFR1 | P11362 | CHEMBL3650 | Kinase |
| Mitogen-activated protein kinase 5 | MAP3K5 | Q99683 | CHEMBL5285 | Kinase |
| Cyclin-dependent kinase 4 | CDK4 | P11802 | CHEMBL331 | Kinase |
| Signal transducer and activator of transcription 6 | STAT6 | P42226 | CHEMBL5401 | Transcription factor |
| Transforming growth factor beta-1 | TGFB1 | P01137 | CHEMBL1795178 | Secreted protein |
| Epidermal growth factor receptor erbB1 | EGFR | P00533 | CHEMBL203 | Kinase |

Target were predicted on strict thresholds of similarity (0.65 for 2D and 0.85 for 3D). AG-PCR; Family A G protein-coupled receptor

**Supplementary Table** 3; PharmMapper Prediction of NSC765598 targets

| **Num Feature** | **Fit** | **Norm Fit** | **Z score** | **Num Hydrophobic** | **Num HB Acceptor** | **Num HB Donor** | **Name** | **Uniplot** |
| --- | --- | --- | --- | --- | --- | --- | --- | --- |
| 3 | 3 | 0.9998 | 0.483331 | 3 | 0 | 0 | MAP kinase-activated protein kinase 2 | P49137 |
| 3 | 2.985 | 0.9952 | 0.641082 | 2 | 1 | 0 | Serine/threonine-protein kinase | mTOR_HUMAN |
| 3 | 2.979 | 0.9929 | 0.377935 | 3 | 0 | 0 | Proto-oncogene serine/threonine-protein kinase Pim-1 | P11309 |
| 3 | 2.961 | 0.987 | 0.53324 | 3 | 0 | 0 | cAMP-dependent protein kinase catalytic subunit alpha | P05132 |
| 3 | 2.946 | 0.9822 | 0.596364 | 2 | 0 | 1 | Mitogen-activated protein kinase 14 | Q16539 |
| 3 | 2.899 | 0.9664 | 0.052795 | 3 | 0 | 0 | Serine/threonine-protein kinase/endoribonuclease IRE1 | P32361 |
| 3 | 2.868 | 0.956 | -0.16496 | 2 | 1 | 0 | Mitogen-activated protein kinase 10 | MK10_HUMAN |
| 3 | 2.797 | 0.9323 | 0.357184 | 2 | 1 | 0 | Cyclin-A2 | CCNA2_HUMAN |
| 3 | 2.739 | 0.9131 | 0.241879 | 2 | 1 | 0 | Cyclin-dependent kinase 5 activator 1 | CD5R1_HUMAN |
| 3 | 2.697 | 0.8989 | -0.26855 | 3 | 0 | 0 | Mitogen-activated protein kinase 1 | MK01_HUMAN |
| 4 | 3.574 | 0.8934 | 1.86815 | 3 | 1 | 0 | Epidermal growth factor receptor | EGFR_HUMAN |
| 4 | 3.311 | 0.8278 | 1.23365 | 3 | 1 | 0 | TGF-beta receptor type-1 | TGFR1_HUMAN |
| 4 | 3.021 | 0.7554 | 0.530478 | 2 | 1 | 1 | Fibroblast growth factor receptor 1 | FGFR1_HUMAN |
| 4 | 2.948 | 0.7369 | 0.1056 | 3 | 1 | 0 | Nitric oxide synthase, inducible | P29477 |
| 4 | 2.919 | 0.7297 | 0.291255 | 3 | 1 | 0 | 3-phosphoinositide-dependent protein kinase 1 | PDPK1_HUMAN |
| 4 | 2.899 | 0.7246 | 0.150642 | 3 | 1 | 0 | B-Raf proto-oncogene serine/threonine-protein kinase | BRAF1_HUMAN |
| 4 | 2.886 | 0.7215 | 0.25161 | 3 | 0 | 1 | Vascular endothelial growth factor receptor 2 | P35968 |
| 5 | 3.603 | 0.7206 | 1.7824 | 4 | 1 | 0 | Hepatocyte growth factor receptor | P08581 |
| 4 | 2.833 | 0.7082 | 0.125856 | 3 | 1 | 0 | Insulin-like growth factor 1 receptor | P08069 |

**Supplementary Table 4**: Enriched KEGG pathways for NSC765598 targets

| Index | Name | matching proteins in your network (labels) | Gene counts | P-value | strenth | Adjusted p-value | Odds Ratio | Combined score |
| --- | --- | --- | --- | --- | --- | --- | --- | --- |
| 1 | hsa05230:Central carbon metabolism in cancer | EGFR,MAP2K1,MTOR,FGFR1 | 4 | 1.516e-9 | 2.3 | 1.556e-7 | 205.13 | 4165.57 |
| 2 | hsa05212:Pancreatic cancer | TGFB1,EGFR,MAP2K1,MTOR | 4 | 2.720e-9 | 2.25 | 2.094e-7 | 177.78 | 3506.27 |
| 3 | hsa05210:Colorectal cancer | TGFB1,EGFR,MAP2K1,MTOR | 4 | 4.747e-9 | 2.19 | 2.924e-7 | 155.04 | 2971.42 |
| 4 | hsa05215: Prostate cancer | EGFR,MAP2K1,MTOR,FGFR1 | 4 | 7.739e-9 | 2.13 | 3.973e-7 | 137.46 | 2567.28 |
| 5 | hsa04066: HIF-1 signaling pathway | EGFR,MAP2K1,NOS2,MTOR | 4 | 8.756e-9 | 2.12 | 3.853e-7 | 133.33 | 2473.80 |
| 6 | hsa05218: Melanoma | EGFR,MAP2K1,FGFR1 | 3 | 8.877e-7 | 2.13 | 0.00002103 | 138.89 | 1935.36 |
| 7 | hsa05214: Glioma | EGFR,MAP2K1,MTOR | 3 | 0.000001005 | 2.16 | 0.00002210 | 133.33 | 1841.44 |
| 8 | hsa04926: Relaxin signaling pathway | TGFB1,EGFR,MAP2K1,NOS2 | 4 | 2.530e-8 | 2 | 9.742e-7 | 102.56 | 1794.08 |
| 9 | hsa05231: Choline metabolism in cancer | EGFR,MAP2K1,MTOR | 3 | 0.000002328 | 2.0 | 0.00004217 | 101.01 | 1310.17 |
| 10 | hsa04012: ErbB signaling pathway | EGFR,MAP2K1,MTOR | 3 | 0.000001468 | 2.07 | 0.00002826 | 117.65 | 1580.19 |
|  |  |  |  |  |  |  |  |  |

**Supplementary Table 5**: Enriched GO Biological process for NSC765598 targets

| #term ID | term description | observed gene count | background gene count | strength | false discovery rate | matching proteins in your network (labels) |
| --- | --- | --- | --- | --- | --- | --- |
| GO:1903800: positive regulation of production of miRNAs involved in gene silencing by miRNA | | 3 | 8 | 3.09 | 4.69E-06 | TGFB1,EGFR,MAP2K1 |
| GO:0007435: salivary gland morphogenesis | | 3 | 28 | 2.54 | 1.29E-05 | TGFB1,EGFR,FGFR1 |
| GO:0045860:positive regulation of protein kinase activity | | 5 | 517 | 1.5 | 1.29E-05 | TGFB1,EGFR,MAP2K1,MTOR,FGFR1 |
| GO:0050679:positive regulation of epithelial cell proliferation | | 4 | 178 | 1.86 | 1.54E-05 | TGFB1,EGFR,MTOR,FGFR1 |
| GO:1905207:regulation of cardiocyte differentiation | | 3 | 44 | 2.35 | 2.29E-05 | TGFB1,EGFR,MTOR |
| GO:0043406:positive regulation of MAP kinase activity | | 4 | 264 | 1.69 | 3.20E-05 | TGFB1,EGFR,MAP2K1,FGFR1 |
| GO:0048568:embryonic organ development | | 4 | 417 | 1.5 | 8.89E-05 | TGFB1,EGFR,MAP2K1,FGFR1 |
| GO:0010634:positive regulation of epithelial cell migration | | 3 | 136 | 1.86 | 0.00016 | TGFB1,MTOR,FGFR1 |
| GO:0010001:glial cell differentiation | | 3 | 154 | 1.8 | 0.00021 | TGFB1,EGFR,MAP2K1 |
| GO:0070374:positive regulation of ERK1 and ERK2 cascade | | 3 | 196 | 1.7 | 0.00031 | TGFB1,EGFR,MAP2K1 |


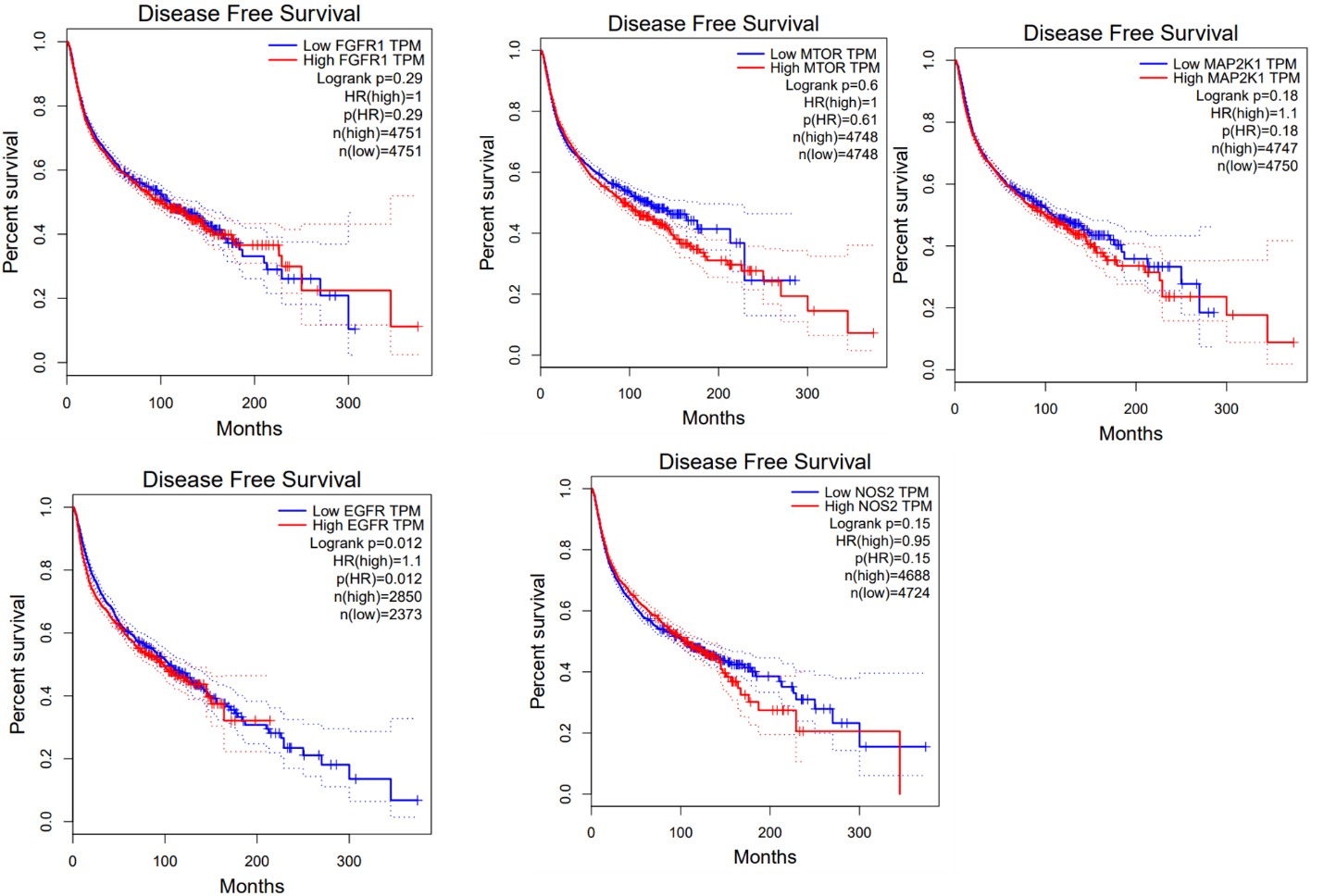


**Supplementary Figure 1**: Disease free survival of EGFR, mTOR, NOS2, TGFB1, MAP2K1, and FGFR1


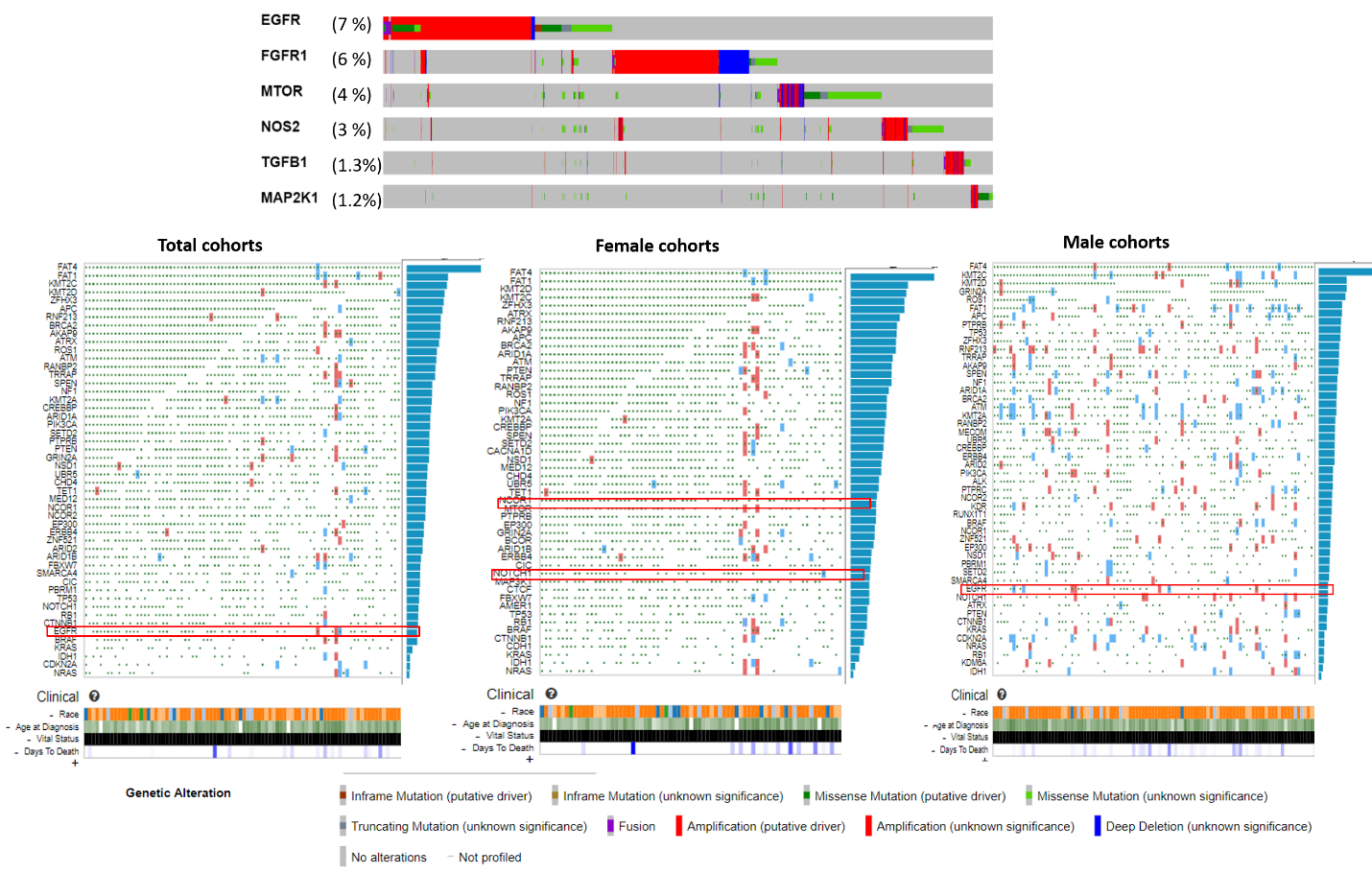


**Supplementary Figure 2**: Frequency of genetic alterations in EGFR, mTOR, NOS2, TGFB1, MAP2K1, and FGFR1


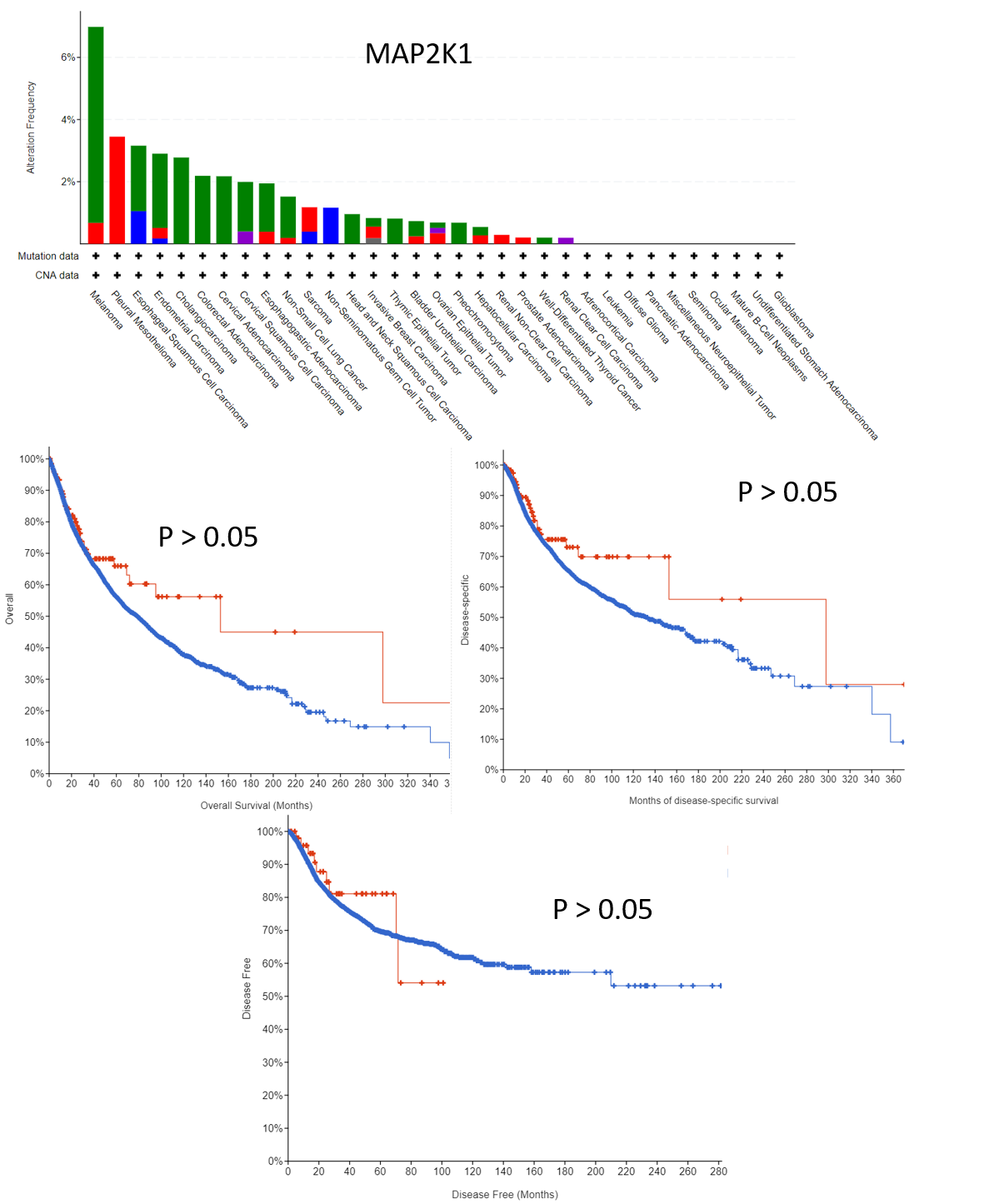


**Supplementary Figure 3**: Frequency of genetic alterations and prognosis relevance of MAP2K1


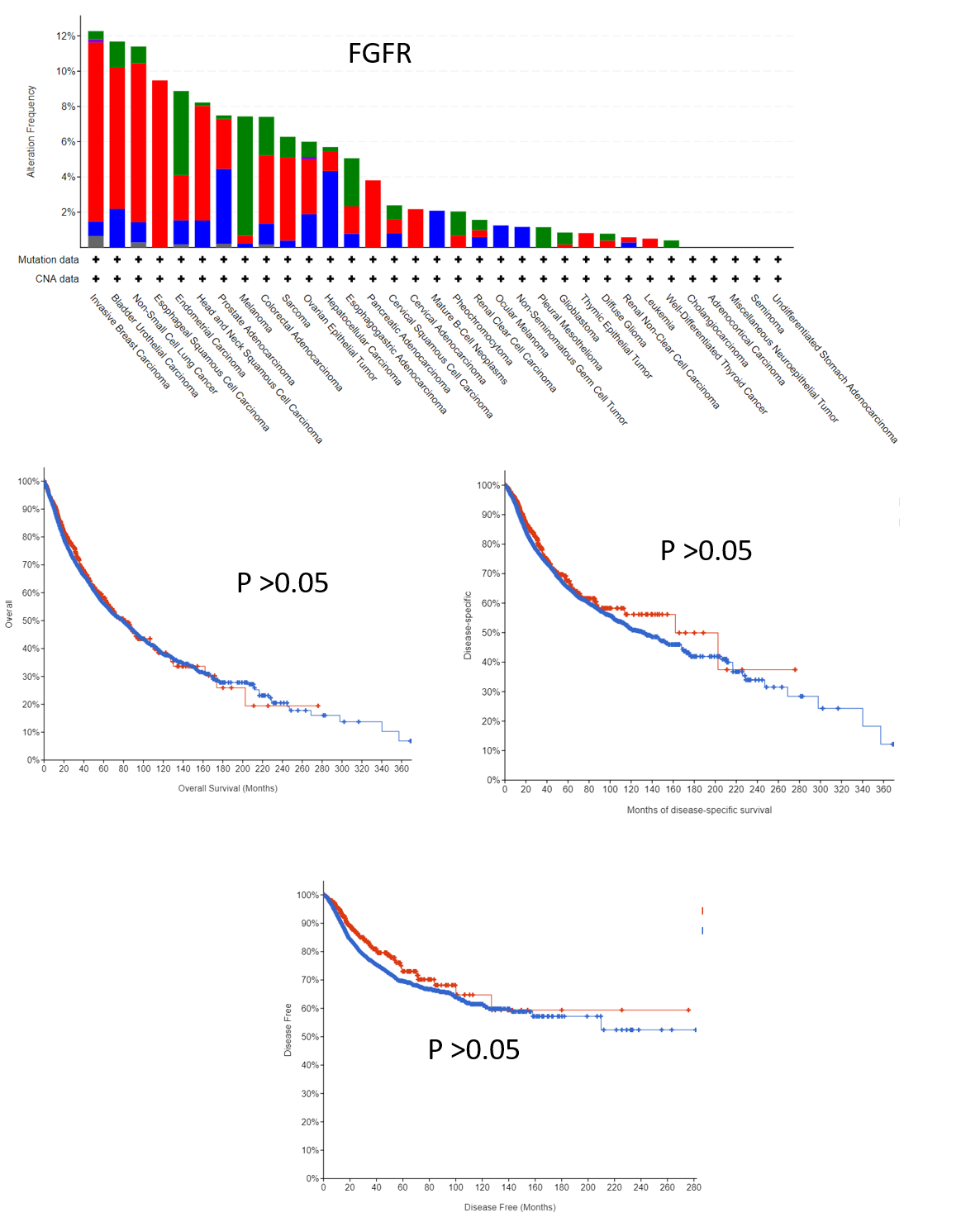


**Supplementary Figure 4**: genetic alterations of FGFR in different cancer and survival analysis

**
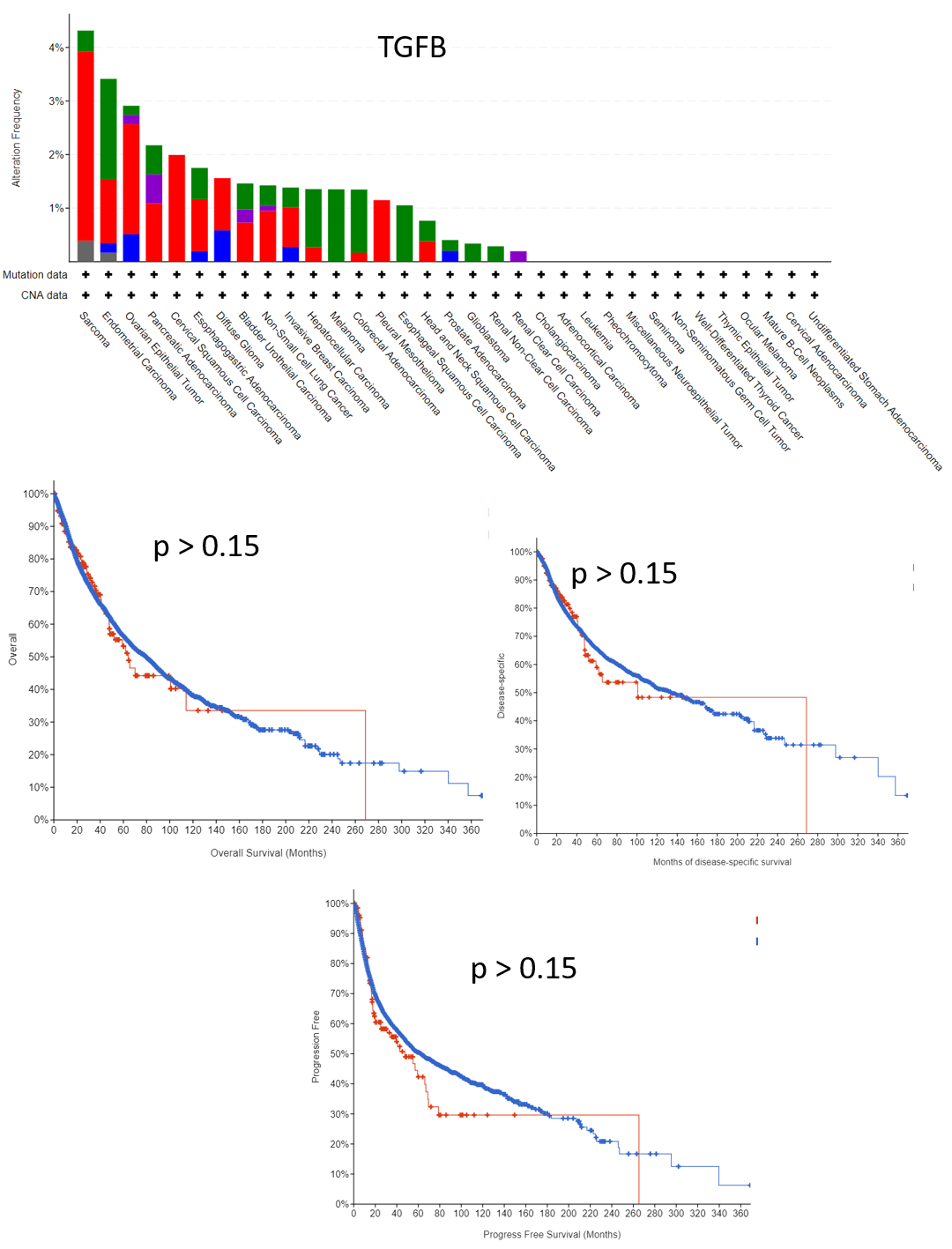
**

**Supplementary Figure 5**: genetic alterations of TGFB1 in different cancer and survival analysis

**
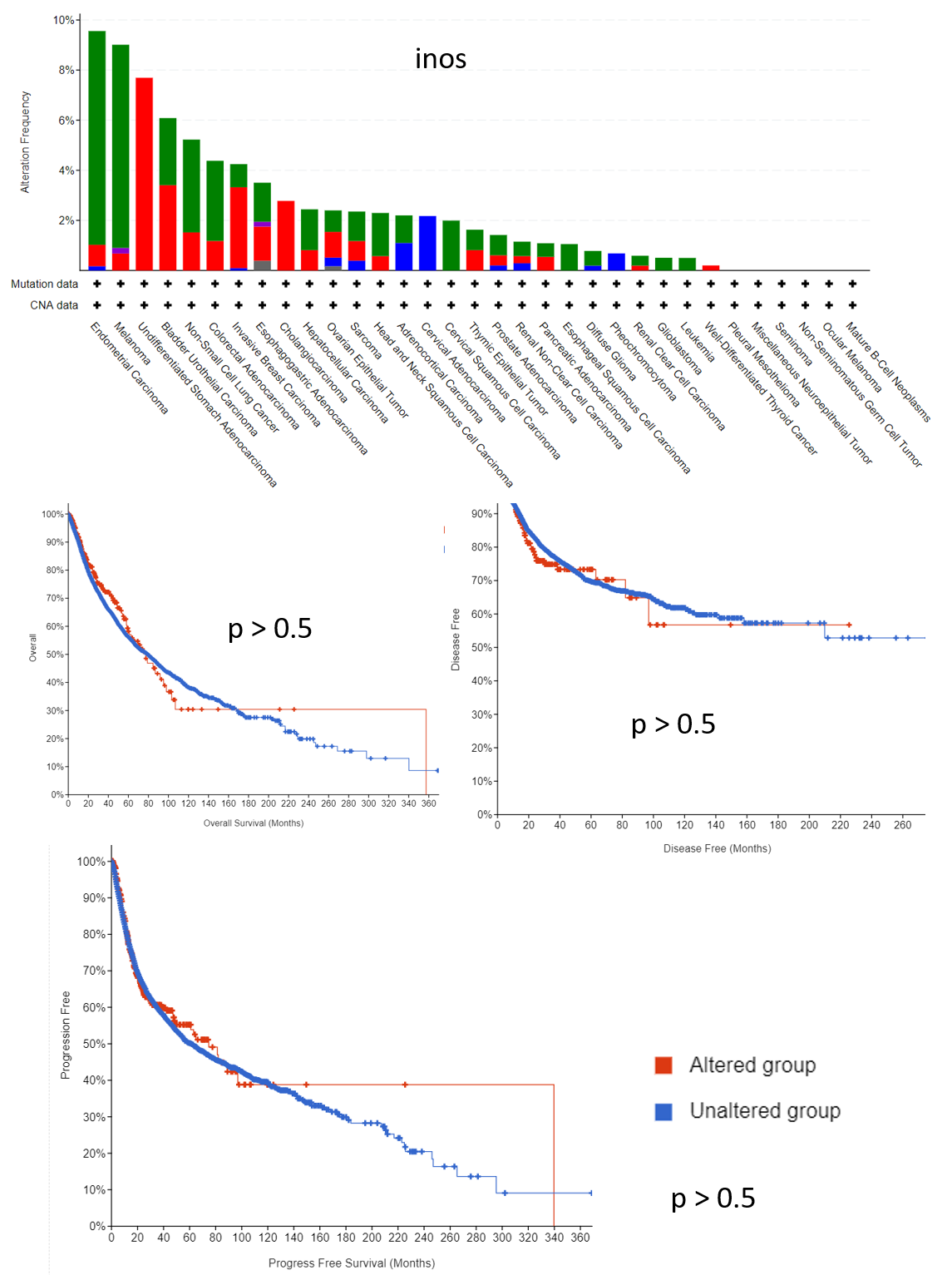
**

**Supplementary Figure 6**: genetic alterations of TGFB1 in different cancer and survival analysis

**
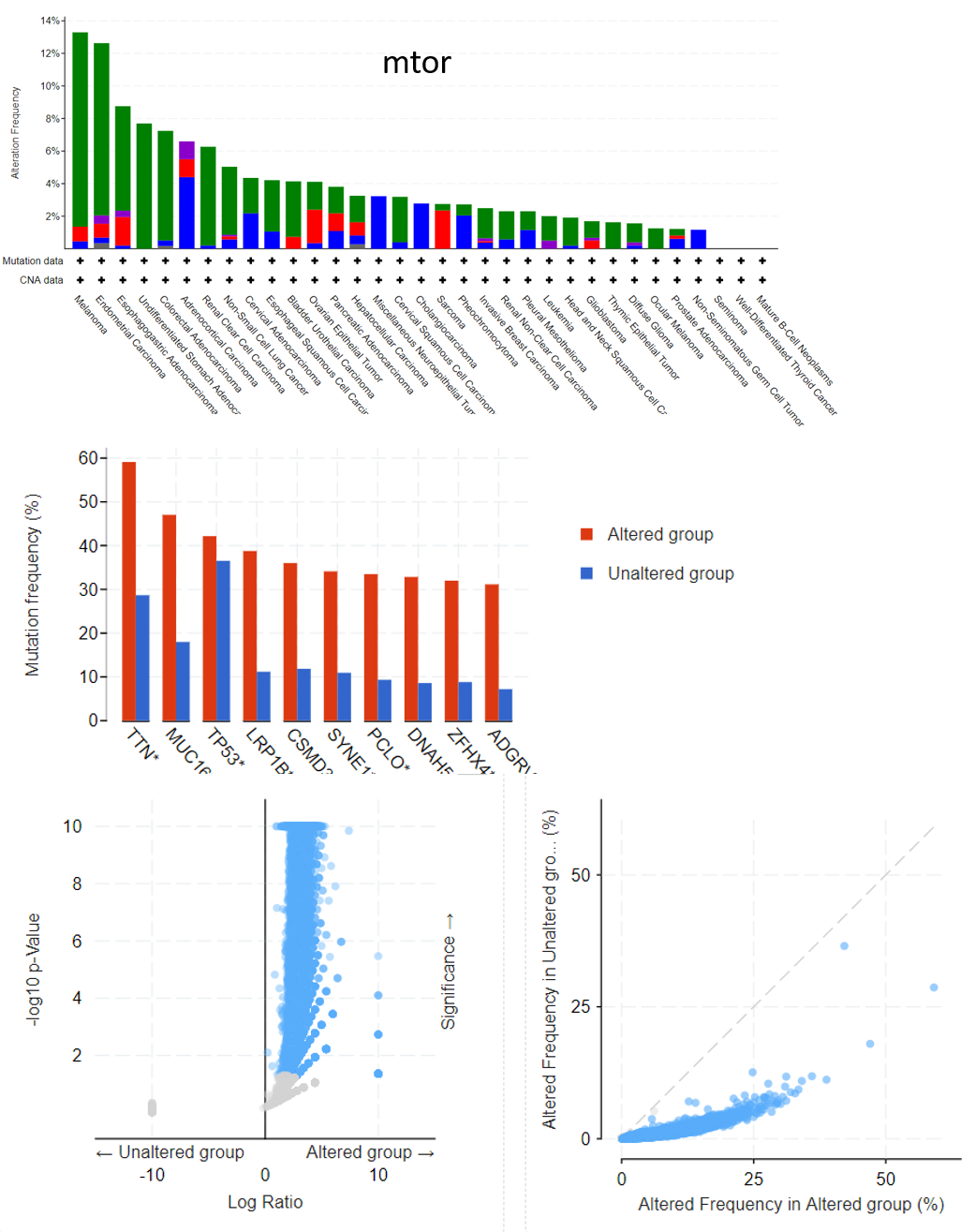
**

**Supplementary Figure 7**: genetic alterations of TGFB1 in different cancer
